# Comparison of tertiary structures of proteins in protein-protein complexes with unbound forms suggests prevalence of allostery in signalling proteins

**DOI:** 10.1186/1472-6807-12-6

**Published:** 2012-05-03

**Authors:** Lakshmipuram S Swapna, Swapnil Mahajan, Alexandre G de Brevern, Narayanaswamy Srinivasan

**Affiliations:** 1 Molecular Biophysics Unit, Indian Institute of Science, Bangalore, 560012, India; 2 Univ de la Réunion, UMR_S 665, F-97715, Saint-Denis, France; 3 INSERM, U 665, Saint-Denis, F-97715, France; 4 INSERM, U 665 DSIMB, Paris, F-75739, France; 5 Univ Paris Diderot, Sorbonne Paris Cité, Paris, F- 75739, France; 6 INTS, F-75739, Paris, France

## Abstract

**Background:**

Most signalling and regulatory proteins participate in transient protein-protein interactions during biological processes. They usually serve as key regulators of various cellular processes and are often stable in both protein-bound and unbound forms. Availability of high-resolution structures of their unbound and bound forms provides an opportunity to understand the molecular mechanisms involved. In this work, we have addressed the question “What is the nature, extent, location and functional significance of structural changes which are associated with formation of protein-protein complexes?”

**Results:**

A database of 76 non-redundant sets of high resolution 3-D structures of protein-protein complexes, representing diverse functions, and corresponding unbound forms, has been used in this analysis. Structural changes associated with protein-protein complexation have been investigated using structural measures and Protein Blocks description. Our study highlights that significant structural rearrangement occurs on binding at the interface as well as at regions away from the interface to form a highly specific, stable and functional complex. Notably, predominantly unaltered interfaces interact mainly with interfaces undergoing substantial structural alterations, revealing the presence of at least one structural regulatory component in every complex.

Interestingly, about one-half of the number of complexes, comprising largely of signalling proteins, show substantial localized structural change at surfaces away from the interface. Normal mode analysis and available information on functions on some of these complexes suggests that many of these changes are allosteric. This change is largely manifest in the proteins whose interfaces are altered upon binding, implicating structural change as the possible trigger of allosteric effect. Although large-scale studies of allostery induced by small-molecule effectors are available in literature, this is, to our knowledge, the first study indicating the prevalence of allostery induced by protein effectors.

**Conclusions:**

The enrichment of allosteric sites in signalling proteins, whose mutations commonly lead to diseases such as cancer, provides support for the usage of allosteric modulators in combating these diseases.

## Background

Protein-protein interactions participate in myriad processes of the cell such as replication, transcription, translation, signal transduction, immune response, metabolism, membrane-associated processes and development (e.g., [1-4]). Protein-protein interactions offer an excellent way of combining its limited working parts, the proteins, to achieve large functional diversity using a limited genetic repertoire [5]. Abnormal interactions between proteins within the cell or from pathogens cause many human diseases [6]. Protein binding can also elicit an allosteric response. Allostery is an integral and pervasive mechanism employed by nature to modulate cellular processes [7-11]. It serves as a key mechanism for obtaining fine-tuned regulation in several cellular processes – from metabolic pathways, signalling systems [12] to gene regulation [13]. Functional modulation is achieved either by enhancing (positive co-operativity) or decreasing (negative co-operativity) levels of function. The effect at target site can be varied, e.g., activation of catalysis, regulation of ligand-binding, control of complex formation[9].

Given their importance, several high-throughput interaction assays [14,15], such as yeast two-hybrid and tandem affinity purification, have been developed to supplement the dataset of protein-protein interactions from low-throughput methods [16,17]. However, such large-scale experimental methods suffer from high false-positive rates [18]. The gold standard for protein-protein interactions is usually a dataset of complexes of interacting proteins solved using X-ray crystallography [19-21]. Although it is a much smaller and incomplete dataset in comparison to high-throughput protein-protein interaction datasets, it is reliable and enables mapping of interaction regions and structural changes which accompany interactions. Several derived databases provide protein-protein interaction datasets in various easy to –study and –use formats. SCOPPI [22], iPfam [23], SNAPPI-DB [24], 3D Complex [25], InterEvol [26] and ProtCID [27] are some of the available 3D structural databases of protein-protein complexes.

Protein-protein interactions can be classified into different kinds [28]: homo-oligomers and hetero-oligomers; obligate and non-obligate complexes; permanent and transient complexes. Non-obligate complexes form an important class since they serve as key regulators in maintaining and regulating cellular homeostasis [29-31]. They are also valuable from the viewpoint of structural biology since both the unbound and bound forms can be crystallized owing to their stability. Several such structures have been solved by various groups and deposited in the Protein Data Bank (PDB) [32]. An invaluable non-redundant dataset of structures of both the interacting partners solved in unbound and bound form has been collated, curated and updated by Weng and colleagues [33,34]. The ComSin database provides a unique collection of structures of proteins solved in unbound and bound form, targeted towards disorder–order transitions [35].

Earlier studies of structures of protein-protein complexes using both the unbound and bound form of proteins reveal that proteins undergo changes in their structure upon binding. Betts and Sternberg [36] were the first to compare the bound and unbound forms using a dataset of 39 complexes. Martin et al. [37,38] analyzed a dataset of 83 complexes in terms of local structural variations. The alterations in structure as a result of protein-protein interactions manifest either as a rigid-body shift of a segment or as a conformational change from one secondary structural form to another [39]. The extent of conformational change observed at the interface upon binding prompted several studies to understand and predict these changes [40-42]. Such studies aim to improve protein-protein docking methods [43] and help in the accurate docking of protein-protein interactions, which can be used to understand the mechanism of functioning of the complex or design inhibitors.

In this work, we have used a curated and non-redundant dataset of 76 protein-protein complexes, solved using X-ray crystallography in high resolution in both unbound and bound form, to address questions about the nature, extent and location of structural changes upon binding. We noticed that, in addition to changes in the interface, possibly allosteric changes causing structural alteration occur in about half of the complexes, indicating a much higher prevalence of this phenomenon caused due to protein binding than appreciated before.

## Results

### Proteins bound to other proteins undergo larger structural changes than unliganded proteins

Structural change observed in different forms of a protein could be due to experimental artifacts [44], intrinsic flexibility [45] or due to a biologically important external perturbation [46], such as ligand binding or post-translational modification. To differentiate structural changes potentially related to protein-protein interactions from those which are artefacts, we compared variations occurring in the dataset of protein-protein complexes with two control datasets (see Additional file 1: Table S1). The first control set (named Control – Rigid) consists of 50 structures, solved at a resolution ≤2.5 Å, of two fairly rigid and extensively studied proteins: bovine ribonuclease A and sperm whale myoglobin, and provides an indicator of co-ordinate uncertainties. The second control set (named Control – Monomer) consists of a non-redundant set of 95 clusters of structures of monomers, also solved at a resolution ≤2.5 Å, which serve as a heterogeneous set since this dataset contains both rigid and flexible proteins, thus serving as a control set for understanding intrinsic flexibility. The main dataset of our study named PPC (Protein-protein complexes) is an extensively curated dataset of non-obligatory proteins with their 3-D structures solved in both unbound and bound forms (Additional file 2: Table S2). It consists of 76 non-obligatory complexes representing members of diverse functions (25 enzyme-inhibitor, 11 antigen-antibody and 40 ‘other’ complexes, which largely comprises of signalling proteins). The number of proteins involved in the 76 complexes represent the major SCOP (Structural Classification of Proteins) [47] classes (all α - 32, all β - 84, α/β - 57, α + β - 37). Since the dataset has been pruned to exclude cases with a large percentage of missing residues at the interface, disordered proteins are under-represented. The complexes predominantly involve two-chain interactions and some interactions involving three chains, in which two chains are considered as a single entity (for example, in the case of light and heavy chains of antibody and in the case of G_β_-G_γ_ subunits in heterotrimeric G-proteins). The proteins constitute a mixture of single-domain and multi-domain members. Although some of the structures in the PPC dataset are solved at a resolution poorer than 2.5 Å, the highest resolution of the Control-Rigid and Control-Monomer datasets, the magnitude of structural changes captured across the datasets can be compared since 50/76 complexes of the PPC dataset were solved with a resolution ≤2.5 Å. The conclusions of comparison of various parameters capturing structural change in the different datasets, discussed below, remained unaltered when using either the ‘50’ or ‘76’ set of complexes (data not shown). The conclusions described below are for the entire dataset of 76 complexes.

**Figure 1:**
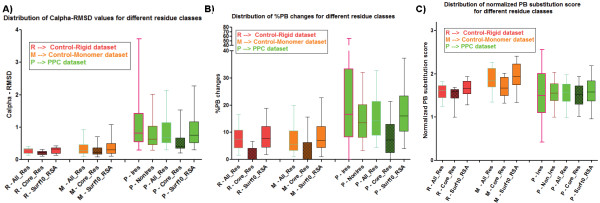
**Distribution of parameters capturing structural change for Control and Test datasets.** Distribution of values for the parameters **A**). Cα RMSD **B**). %PB changes and **C**). PB substitution scores calculated at a per-protein level for Control-Rigid, Control-Monomer and PPC datasets. Buried residues are indicated with filled boxes. Ires - interacting residues; NonIres - non-interacting residues; Core_Res (≤5% RSA) - buried residues; Surf10_RSA (>10% RSA) – surface residues. The figure shows that protein-protein complexes undergo significantly larger structural changes when compared with unliganded forms for all residues types. The p-values for all of the following comparisons performed using Mann–Whitney test indicates statistical significance (*p*-value < 0.0001) : M-All_Res vs. P-All_Res, M-Core_Res vs. P-Core_Res, and M-Surf10_RSA vs. P-Surf10_RSA, for all the 3 parameters. This trend is prominently captured by the parameters Cα RMSD and %PB changes.

Three parameters were used to analyze structural change occurring in the different types of residues (see Methods) in a protein: root mean square deviation (RMSD), %PB change (PBc), PB substitution score (PBSSc). Although RMSD captures the magnitude of structural change, it does not distinguish the type of structural change – i.e. rigid body movement (or) conformational change. The use of Protein Blocks (PBs) enables this distinction since small yet significant changes in local conformation of a protein can be captured using PBs. Protein Blocks consists of 16 standard conformational states of pentapeptides [48,49]. PBs can be used to represent precisely backbone conformation of all the protein structures known so far. This efficient design has been employed in several applications, including prediction of long fragments and short loops, and in identifying proteins with similar structures [49,50]. A PB change between the unbound and bound forms for the equivalent residue indicates a conformational change – either subtle or drastic. The % PBs altered between two structures serves as a metric for capturing the extent of structural change (PBc). A substitution matrix derived earlier [50] was used to calculate the magnitude of structural dissimilarity between two structures in terms of their PB changes (namely PBSSc). A lower PBSSc indicates unfavourable changes (i.e. drastic conformational change – for example, a helix to a strand) whereas a higher PBSSc indicates milder conformational changes (for example, change between a curved helix and a linear helix). Analysis of the three parameters revealed that all types of residues (buried, surface, interacting) undergo higher structural change upon binding to another protein than in the unliganded form (Figure 1). These values are calculated at per-protein level for the different classes of residues. RMSD (Figure 1A) and PBc (Figure 1B) clearly showed higher structural variation of protein-bound forms in comparison to the unbound forms whereas PBSSc (Figure 1C) showed a marginal trend. This is because the PB changes could be of two kinds: favourable (high PBSSc) and unfavorable (low PBSSc) and both are represented in the graph. As expected, buried residues showed the least deviation of all the classes and interacting residues the highest change. Buried residues are mostly invariant, as seen from the box plot depicting the distribution of PBc (Figure 1B), where ~50% of the values are zero for the control datasets. Surprisingly, ~90% of buried residues of protein-protein complex structures show at least a single conformational change, as characterized by change in PB (Figure 1B). However, the observed changes are mostly minor. In the rare cases when it is a large change, the residue is seen to have slight exposure to solvent.

In order to distinguish structural variations caused due to protein binding from those occurring due to crystallographic artifacts, the upper bound values corresponding to the Control-Rigid dataset were used as reference for the three parameters (see Additional file 3: Figure S1).

It is observed that the main protein-protein complex dataset comprises of complexes with varying range of interface area and size of proteins (see Additional file 2: Table S2). Therefore, dependence of the parameters capturing structural change for interface area and size of the protein (represented as length of the protein) were analyzed. The analysis indicates that there is slight dependence of RMSD, PBc and PBSSc for interface area buried by the complex whereas the parameters have negligible dependence on the lengths of proteins (see Additional file 4: Figure S2).

Independently, we also captured structural change using all-atom RMSD which includes consideration of sidechain atoms. Although there is expected variation in all-atom RMSDs for interacting residues (see Additional file 5a: Figure S3), there is no profound variation when the Cα RMSDs are compared with corresponding all-atom RMSD values (see Additional file 5b: Figure S3). Therefore, the present analysis is confined to Cα RMSD based comparison in this study.

### Pre-made interfaces predominantly bind to structurally-altered interfaces

**Figure 2:**
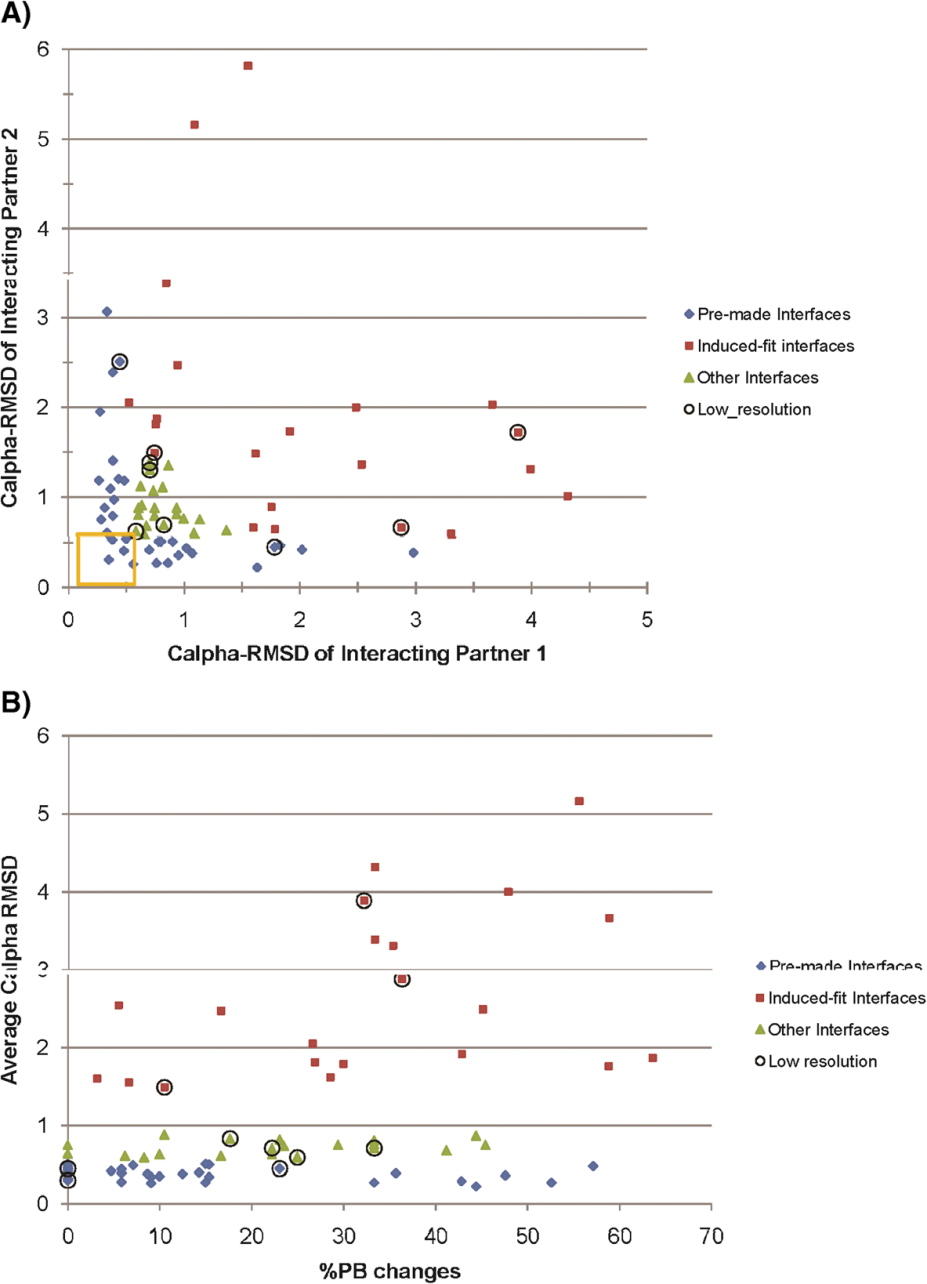
**Characteristics of different types of interfaces.** The three kinds of interfaces are pre-made (blue color), induced-fit (brown) and others (green). **A**). A plot of Cα RMSD for the pair of interacting partners is shown. Completely pre-made interfaces are enclosed in a yellow square. **B**). The extent of conformational change for the three kinds of interfaces is shown. The graphs plot only the majority of the points (Cα RMSD ≤6 Å) for the sake of clarity. This figure illustrates that **A**). Pre-made interfaces largely bind to induced-fit interfaces, and** B**). Although pre-made interfaces show small magnitude of structural change, the extent of conformational change they undergo is comparable to that observed in induced-fit/other interfaces. For both sets, points corresponding to complexes solved at a resolution >2.5 Å are encircled.

The extent of structural change at both the interfaces of various complexes has been assessed. Proteins are classified into three categories based on the extent of structural change at the interfaces of various complexes: pre-made, induced-fit, and other. Interfaces exhibiting Cα RMSD of <0.5 Å (which is the maximum deviation between any two proteins of the Control-Rigid dataset) are considered pre-made, while those with Cα RMSD of >1.5 Å are considered induced-fit. The interfaces showing structural changes between these two values are classified in the ‘other’ category. We identify 33 pre-made interfaces, fitting the lock-and-key hypothesis proposed to explain protein-ligand binding [51]. Such a large number is surprising since the proteins would always be primed for interaction. Nature’s regulatory control of the primed pre-made interfaces appears to be achieved via its partner interface. It appears that although one of the interfaces is pre-made, the other interface undergoes substantial changes (Figure 2A, blue coloured points) in the final stable bound form (sometimes >1.5 Å Cα RMSD, the cut-off for identifying ‘induced-fit’ interfaces). 9 protein-protein complexes, 5 from ‘Other’ category and 4 from Enzyme-Inhibitor category, show this behaviour. 17 of the 33 pre-made interfaces had almost no PB changes, implying near complete absence of conformational changes. However, the interaction seems to be modulated by the structural changes occurring in the partner interface. In 14/17 of these cases, the partner’s PBc is >15%; in three cases it is >40% (Average PBc is 26 ± 14). Only five of the 33 pairs seem to be pre-made in both interfaces. However, inspection of PBc for these ‘pre-made interfaces’ revealed that there are substantial conformational changes of smaller magnitude captured using PBs which use atomic positions of N, C and O atoms apart from Cα as opposed to Cα-based RMSD (see Figure 2B). For instance, the complex of cytochrome C peroxidise and iso-1-cytochrome C forms a pre-made interface (see Additional file 6A: Figure S4), with Cα RMSD of 0.37 Å and 0.31 Å for the interacting proteins. However, the conformations of side chain positions of interface residues changes drastically in 3/18 interface residues. This example supports the hypothesis that almost all interacting partners undergo changes upon binding, even if one of the interfaces is pre-made, and PBs help in identifying subtle changes than classical RMSD measures. In essence, there are no ‘completely pre-made’ interfaces.

Usually, interfaces with an average Cα RMSD of ≥1.5 Å showed substantial changes at interface, exemplifying the concept of induced-fit hypothesis [52] for formation of protein-protein complexes (see Additional file 6B: Figure S4). 35 interfaces with average Cα RMSD of ≥1.5 Å are found. Of these predominantly altered interfaces, 10 are partners of pre-made interfaces, 4 are partners of like-wise induced-fit interfaces and the rest have values in between (see Additional file 6C,D: Figure S4).

Comparison of the structural change in terms of Cα RMSD and normalized PB substitution score can help in distinguishing cases of rigid body movements from conformational changes. Induced-fit interface regions with 0% PB change at interface can be considered to have rigid-body movements (see Additional file 7A: Figure S5). However, since PBs are very sensitive to backbone torsion angle changes, two very similar PBs will also be considered as PB changes. Therefore, normalized PB substitution score is a more pertinent metric to grade the local conformational change (see Additional file 7B: Figure S5).

**Figure 3:**
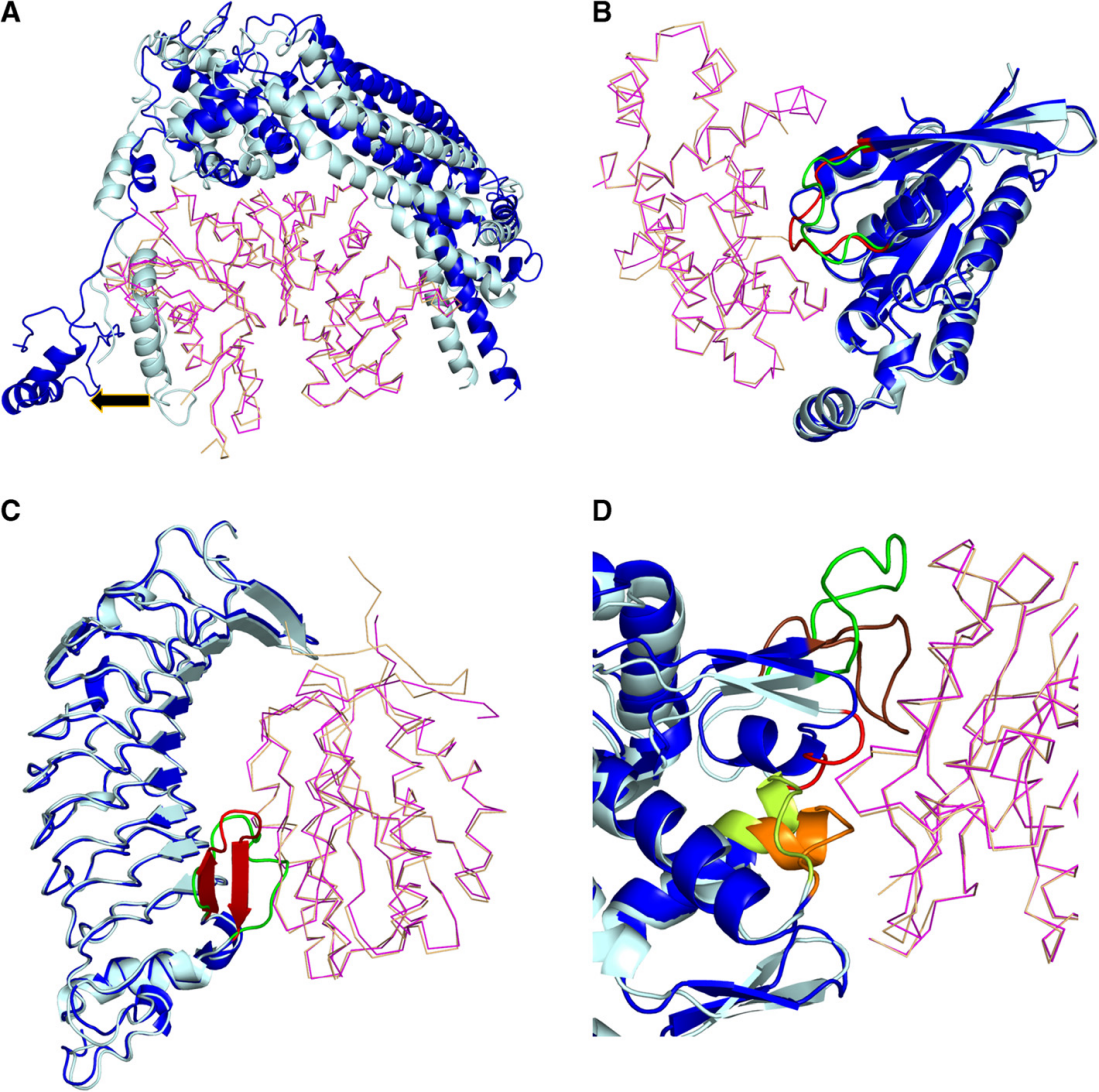
**Structural changes observed in interfaces.** Protein undergoing change is shown as cartoon, with unbound form in light cyan and the bound form in blue, and its partner as a ribbon, with unbound form in light orange and bound form in magenta. Direction of movement is indicated as black arrow. **A**) Large (~10 Å Cα RMSD) *moving out* to avoid steric clash (alpha actin & BNI1 protein; 1Y64). **B**). Movement to optimise interaction with partner (GTP binding protein & Rho GTPase activating protein; 1GRN). **C**). Conformational change accompanied by movement mainly to avoid steric clashes with the partner (Glycoprotein Ib alpha & von Willebrand factor; 1 M10). In B) and **C**), the region of interest is colored green and red in the unbound and bound forms, respectively. **D**). An interface (actin & deoxyribonuclease I; 1ATN) where certain region moves away to avoid steric clash (colored in red), some region undergoes conformational change with movement to optimise an interaction (depicted in green for the unbound form and brown for the bound form) and another region undergoes rigid body movement to optimise its interaction (colored in lemon yellow in the unbound form and orange in the bound form). All the figures containing protein structures were generated using PyMOL [77].

Large structural changes could result for different reasons such as to avoid steric clashes and/or optimize binding. In some cases, global changes in the molecule (both interface and non-interacting surface RMSDs are ≥1.5 Å) are observed. These complexes either move out (Figures 3A, Additional file 8E: Figure S6) (or) move in (Additional file 8B: Figure S6) to relieve steric clashes/optimize binding, respectively. In most cases, changes were localized at the interface, comprising of rigid-body movements (Additional file 8C: Figure S6) or conformational changes (Additional file 8D: Figure S6) or conformational changes with movement (Additional file 8A: Figure S6), to mainly optimize binding (Figure 3B) or relieve steric clashes (Figure 3C) or both (Figure 3D). Local rearrangements at the interface are identified based on the normalization-based metric (see Methods section). This criterion allows us to identify interfaces with proportionately larger localized changes at the interface although the magnitude is smaller (≤1.5 Å) (Additional file 6D: Figure S4). In cases where the change is larger (≥2 Å), rearrangement seems to be mainly targeted at avoiding steric clashes. In cases where the change is moderate (1.5 Å ~ 2 Å), the rearrangements appear to be mostly for proper optimization of interface.

### Non-interacting regions away from the interface undergo substantial structural changes on binding

**Figure 4:**
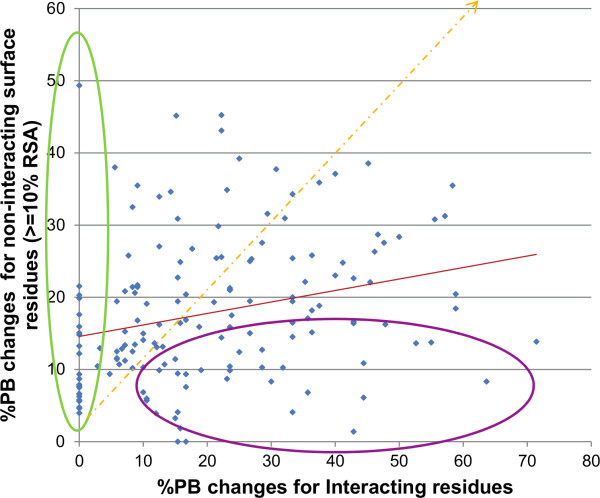
**Scatter plot of PBc for interacting residues vs. rest of surface residues for PPC dataset.** Proteins showing higher proportion of PB changes at the interface are encircled in purple whereas proteins showing PB changes in the non-interacting surface region when the interacting region remains unaltered are encircled in green. This plot reveals the existence of several protein-protein complexes which exhibit substantial conformational changes at non-interacting surface regions even though the interface region is largely unmodified (shown in green circle).

In general, interacting residues undergo larger structural change than non-interacting surface residues (≥10% residue surface accessibility (RSA)). Comparison of the three parameters quantifying structural changes studied for individual proteins showed that this trend holds true even in these cases (Figure 4, Additional file 9: Figure S7). Indeed, interacting regions need to undergo rearrangement to form an optimal fit. The general trend of comparatively larger changes at interface regions was seen for RMSD and PBSSc (see Additional file 9: Figure S7). However, the parameter PBc provided a new insight, highlighting cases with almost no conformational change at the interface but with considerable change in the rest of the surface (Figure 4, green ellipse). This emphasizes that there exist complexes in which non-interacting regions undergo structural variation upon binding even though the interface remains largely unchanged. 6% of the complexes exhibited 10%-25% PBc and one case showed 50% PBc in the non-interacting surface region (Figure 4).

Although interacting regions undergo large structural changes in comparison to the rest of the surface, about one-half of the cases in the PPC dataset reveal large changes away from the interface (Figure 4, Tables 1 &2). PB changes in non-interfacial regions can be divided into two cases. *(i)* Change in non-interacting regions even when there are almost no changes in interacting regions (*n* = 22) (Table 1). (*ii*) Change in non-interacting regions accompanying changes in interacting regions (*n* = 12) (Table 2). The two categories combine to provide a dataset of 34/76 complexes exhibiting substantial structural change in non-interfacial surface regions. Interfaces represented in the first case can be considered as pre-made since no PB change is observed on complexation. As expected, the partner protein for these interfaces exhibited much larger change at the interface.

**Table 1:** Features of proteins with substantial structural change in non-interacting regions and no/moderate change at interface

	**PDB code**		**Cα RMSD**			**Normalized PB substitution score**		
Protein	Bound	Unbound	IR*	NIR_PBc*	Difference	IR*	NIR_PBc*	Difference
					(NIR_PBc – IR)			(NIR_PBc – IR)
PIP3 kinase (O)	1HE8_r	1E8Z	0.49	8.45	7.96	1.25	-1	-2.25
HISF protein (O)	1GPW_r	1THF	0.68	8.10	7.42	1.68	-1.31	-2.99
UCH-L3 (O)	1XD3_r	1UCH	1.30	5.11	3.80	1.35	-1.05	-2.4
Son of Sevenless (O)	1BKD_r	2II0	1.33	4.91	3.57	1.39	-0.82	-2.21
TGF-beta (O)	1KTZ_r	1TGK	0.66	4.41	3.75	2.1	-0.95	-3.05
HPr kinase C-ter domain (E)	1KKL_r	1JB1	1.76	4.00	2.24	1.95	-0.98	-2.93
Cystatin (E)	1YVB_l	1CEW	0.63	3.85	3.21	2.43	-1.24	-3.67
DH/PH domain of TRIO (O)	2NZ8_r	1NTY	1.33	3.77	2.43	1.25	-0.78	-2.03
Actin (O)	2BTF_r	1IJJ	1.08	3.77	2.68	1.76	-0.81	-2.57
Alpha-1-antitrypsin (E)	1OPH_r	1Q1P	0.92	3.50	2.58	1.35	-1.39	-2.74
TGFbeta receptor (O)	1B6C_l	1IAS	1.17	3.14	1.96	1.28	-1.04	-2.32
Vitamin D binding protein (O)	1KXP_l	1KW2	1.53	3.11	1.57	1.66	-0.77	-2.43
TolB (O)	2HQS_r	1CRZ	1.37	3.04	1.67	2	-0.81	-2.81
RCC1 (O)	1I2M_l	1A12	0.40	3.00	2.60	2.43	-0.99	-3.42
Sporulation response factor B (O)	1F51_r	1IXM	0.78	2.70	1.92	1.1	-0.97	-2.07
Ran GTPase (O)	1A2K_l	1QG4	0.33	2.57	2.24	2.56	-1.03	-3.59
HEW lysozyme (A)	1BVK_l	3LZT	0.31	2.57	2.25	2.86	-1.06	-3.92
Transferrin receptor ectodomain (O)	1DE4_l	1CX8	0.94	2.51	1.57	1.04	-1.16	-2.2
Anthrax toxin receptor (O)	1T6B_l	1SHU	0.27	2.32	2.05	2.15	-0.75	-2.9
Xylanase inhibitor (E)	2B42_r	1T6E	0.33	2.25	1.91	2.06	-0.95	-3.01
Fab (A)	1E6J_r	1E6O	0.75	1.98	1.22	2.48	-0.73	-3.21
Complement C3 (O)	1GHQ_r	1C3D	0.21	1.58	1.36	1.92	-0.69	-2.61

*The abbreviations used are: IR - Interface regions, NIR_PBc – Non-interacting regions with PB change, l – ligand (smaller of the two proteins in the complex), r – receptor (bigger of the two proteins in the complex), E – enzyme-inhibitor complex, A – Antigen-antibody complex, O – Other complexes.

The proteins are listed in decreasing order of average Cα RMSD of NIR_PBc values. The average Cα RMSD of IR and NIR_PBc is 0.89 and 3.75, respectively. The normalized PB substitution score for IR and NIR_PBc values is 1.78 and -0.98, respectively.

**Table 2:** Proteins with substantial structural change in non-interacting regions and interfacial regions

	**PDB code**			**Cα RMSD**		**Differences from global RMSD**		**Normalized PB substitution score**	
Protein	Bound	Unbound	IR*	NIR_PBc*	Global RMSD	IR* – Global RMSD	NIR_PBc* – Global RMSD	IR*	NIR_PBc*
Arf1 GTPase (O)	1R8S_r	1HUR	5.19	5.42	3.02	2.17	2.40	0.79	-0.67
Ras GTPase (O)	1BKD_l	1CTQ	4.39	4.43	2.21	2.18	2.22	0.63	-1.82
CDK2 kinase (E)	1FQ1_l	1B39	4.39	4.26	2.04	2.35	2.22	1.31	-1
FC fragment of human IgG 1 (A)	1E4K_r	2DTQ	3.17	3.77	2.18	0.99	1.59	0.95	-0.99
Ran GTPase (O)	1I2M_r	1QG4	3.27	3.51	1.91	1.36	1.60	0.58	-0.8
Cystein protease (E)	1PXV_r	1X9Y	3.99	3.47	1.48	2.51	1.99	1.2	-1.04
Rab21 GTPase (O)	2OT3_l	1YZU	4.65	3.30	1.71	2.94	1.59	0.1	-0.6
CDC42 GTPase (O)	1GRN_r	1A4R	2.66	2.97	1	1.66	1.97	1.31	-0.96
Rac GTPase (O)	2NZ8_l	1MH1	3.87	2.68	1.17	2.70	1.51	-0.03	-1.18
Actin (O)	1ATN_r	1IJJ	6.09	2.58	1.54	4.55	1.04	0.97	-0.59
Rac GTPase (O)	1I4D_l	1MH1	2.31	2.52	0.81	1.50	1.71	1.15	-0.65
Glycoprotein IB-alpha (E)	1M10_l	1MOZ	3.95	2.12	0.89	3.06	1.23	0.91	-0.95

*The abbreviations used are: *IR* - Interface regions, *NIR_PBc* – Non-interacting regions with PB change, *l* – ligand (smaller of the two proteins in the complex), *r* – receptor (bigger of the two proteins in the complex), *E* – enzyme-inhibitor complex, *A* – Antigen-antibody complex, *O* – Other complexes.

The proteins are listed in decreasing order of average Cα RMSD of NIR_PBc values. The average Cα RMSD of IR and NIR_PBc is 3.99 and 3.42, respectively. The average difference in RMSD values for IR and NIR_PBc from global RMSD are 2.33 and 1.75, respectively. The normalized PB substitution score for IR and NIR_PBc values is 1.737 and −0.95, respectively.

**Figure 5:**
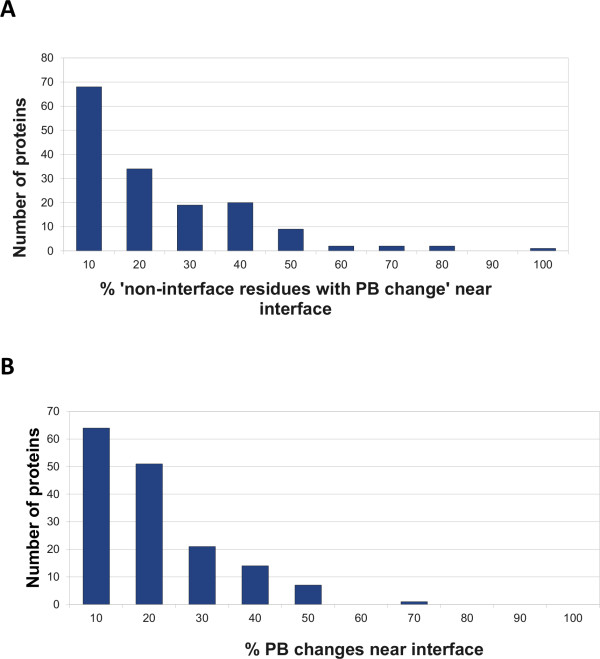
**Distribution of non-interacting residues with PB change. A**). Histogram of “% of ‘residues nearby interface’ in a protein undergoing PB change” is plotted. **B**). Histogram of “% of non-interacting residues with PB change” which are near to interface is plotted. The upper-bound value for every range is indicated as the label on x-axis. This figure reveals that most of the conformational changes occurring in the non-interacting surface regions are not near the interface.

Changes occurring in the non-interfacial regions are classified as near the interface region or away from the interface. All non-interfacial residues in a protein which are within a distance of ≤6 Å Cα distance from any of the interacting residues were considered as ‘residues nearby interface’, since they occur in the vicinity of the interfacial residues and are important for the formation of the structural scaffold [53]. Figure 5 shows that in most of the proteins, the residues nearby interface do not undergo much change (Mean – 15.19%, Median – 12.31%); the highest peak is at 10%, which means that most of the changes occurred away from the interface. This fact was also confirmed by visual inspection of the structure of the protein-protein complexes.

### Conformational changes occurring away from the interface are potentially allosteric: Literature-based, structure-based and normal mode analysis

To ascertain any known or potential biological relevance for these changes, all the ‘non-interacting regions with PB change’ in the identified proteins were analyzed using following parameters: (i) Crystallographic temperature factor (B-factor). Regions with low flexibility and different conformations are likely to have adopted the particular conformation. Studies report that interacting sites have lower B-factors than rest of the protein surface on average even in the unbound form [54]. (ii) Known functional roles of residues: SITE records listed in PDB files and Catalytic Site Atlas (CSA) [55] were consulted to identify if any of the known functionally important residues for the protein of interest are present in the non-interacting regions with PB change. (iii) Literature survey: Relevant literature of the crystal structures was studied to check for any previously known information about these observed PB changes for each protein. The information gathered from the above sources is listed in Table 3. The B-factor distribution for the non-interacting residues with structural change varied from ‘low’ (normalized B-factor < −1, see Methods) to ‘very high’ (normalized B-factor >3, see Methods) values. Unfortunately, PDB SITE records and CSA did not provide information in most cases. Literature survey, although unable to account for all the structural changes observed in the non-interfacial region, indicates that many of these changes are allosteric (15/34) [56-70].

**Table 3:** Features of non-interacting regions with substantial conformational change upon protein-protein interaction

**Protein**	**B-factor**		**PDB SITE record/Crystal atlas**	**Crystal packing**
	Bound	Unbound		
*Proteins with almost no structural change at the interface*				
PIP3 kinase (O)	Poor resolution and many missing residues		NI	-
HISF protein (O)	*H*	*H*	Catalytic site residues not present in NIR_PBc/IR	-
*UCH-L3 (O)*	*Nh*	*Nh*	*Out of 4 active site residues, one in IR*	*NIR_PBc is near IR and are rigid in unbound form, one of the symmetry-related molecules is situated ~6Å towards this region.*
Son of Sevenless (O)	H	Nh	NI	-
TGF-beta (O)	Missing residues near this region		NI	-
HPr kinase C-ter domain (E)	Resolution of PDBs is 2.8 Å		NI	-
Cystatin (E)	*H*	*H*	NI	-
DH/PH domain of TRIO (O)	Temperature factors for region under consideration abnormally high!		NI	-
*Actin (O)*	*H*	*Not so high*	*4/12 important residues are present in NIR_PBc*	*Region involved in dimerization in unbound form*
*Alpha-1-antitrypsin (E)*	*H*	*H*	*Important residues present in IR*	*-*
*TGFbeta receptor (O)*	*Nh*	*Nh*	*3/5 of active site residues are present in NIR_PBc*	*-*
*Vitamin D binding protein (O)*	*Nh*	*Nh*	*NI*	*NIR_PBc values are close to the ones observed are IR and rigid in unbound form, one of the 4 symmetry-related molecules comes ~7Å towards this region. Another NIR_PBc value is far away from IR and is slightly mobile - the same symmetry-related molecule comes <5Å close to this region!*
*TolB (O)*	*Nh*	*H*	*NI*	*-*
Ran GTPase (O)	Nh (nearby IR), H	H, Nh	NI	-
Sporulation response factor B (O)	Missing residues near this region		NI	*-*
Ran GTPase (O)	H	Nh	One important residue present in IR	-
*HEW lysozyme (A)*	*Nh*	*Nh*	*Catalytic site residues not present in NIR_PBc/IR*	*-*
Transferrin receptor ectodoma in (O)	Poor resolution.		Important residues not present in IR/NIR_PBc	*-*
Anthrax toxin receptor (O)	Missing residues near this region		NI	*-*
Xylanase inhibitor (E)	*H*	*H*	Catalytic site residues not present in NIR_PBc/IR	-
Fab (A)	*H*	*Very high*	NI	-
*Complement C3 (O)*	*Nh*	*Nh*	*Important residues are not present in NIR_PBc/IR*	*-*
*Proteins with substantial structural change at interface*				
*Arf1 GTPase (O)*	*Nh*	*Nh*	*-*	*-*
*Ran GTPase (O)*	*Nh*	*Nh*	*Important residues are present in NIR_PBC/IR*	*-*

We observed that most of the proteins with changes are signalling proteins (17/22 in Case 1: Conformational changes in non-interacting surface regions of proteins with invariant interfaces & 8/12 in Case 2: Conformational changes in non-interacting surface regions of proteins with altered interfaces). Therefore, 25/40 ‘other’ complexes (predominantly signalling proteins) from the PPC dataset show significant structural changes at distal sites, indicating prevalence of this phenomenon in signalling proteins. In contrast, only 7/25 and 3/11 complexes of the enzyme-inhibitor and antigen-antibody classes, respectively, show such changes.

Detailed information about the residue positions and the nature of conformational change observed in the residues possibly forming the target site for all examples of Case 1 and Case 2 are listed in Additional file 10: Table S3 & Additional file 11: Table S4, respectively.

Although literature studies implicate allosteric communication to be the reason for the observed structural changes away from the interface in nearly half of the complexes, we did not get clues for the other cases. Since flexibility of a region is known to be good indicator of functional relevance [45], we used this as a metric to identify the biological relevance of the structural changes in all the cases. Coarse-grained normal mode analysis (NMA) [71] is an effective and widely used method to identify intrinsic dynamics of biomolecules at equilibrium conditions solely based on their 3-D structures. This approach computes all possible vibrational modes in which the molecule can move. Studies show that biologically important functional motions are almost always captured within one or many low-frequency modes, since they require the least energy for conformational transitions [72]. Each mode indicates an intrinsic tendency for collective reconfiguration at particular regions. Coarse grained NMA has been applied to various aspects of structural biology, ranging from prediction of functionally relevant motion in proteins and assemblies, refinement of cryo-EM structures, identification of notable evolutionarily conserved dynamic patterns in protein families, to guiding protein docking to proceed along trajectories deemed to be functionally relevant [72,73]. Specifically, a study of four protein-protein complexes using different variations of NMA to identify the regions and directionality of structural change revealed that these changes correlate with intrinsic motions of the protein in the unbound form [74]. A Gaussian network model (GNM) –based NMA of the unbound proteins in Case 1 and Case 2 sets that contain ‘non-interacting regions with PB change’ with low B-factors in both unbound and bound forms were carried out using oGNM web server, to identify regions exhibiting intrinsic motion, which has largely been observed to correlate with biologically relevant regions [75]. A summary of the normal mode analysis results are presented in Table 4. NMA indicates that structural changes away from the interface in some of the complexes are functionally relevant. A study by Chennubhotla and Bahar indicates that a method that combines information theoretic concepts with normal mode analysis can be used to determine the communication mechanisms encoded in the structural topology of the protein [76]. Based on these studies, allostery, which has already been observed in 15/34 complexes according to literature reports, appears to be the most likely mechanism to explain the structural changes occurring at regions away from the interface arising from protein binding.

**Table 4:** Results of normal mode analysis for proteins with ‘non-interacting regions with PB change’

**PDBcode**	**Region of interest**	**Mobile/Rigid?**	**Whole/Embedded**	**Mode**
*Cases with substantial change in non-interacting residues whereas interacting region has very less change*				
2HQS:A	Interface region	Highly mobile	Whole	5
	NIR_PBc far away (mainly 86-91)	Highly mobile	Whole	3
1GHQ:A	Interface region	Highly mobile	Whole	1
	NIR_PBc far away (264-274)	Partially mobile	Embedded	1
1OPH:A	Interface region	Highly mobile	Whole	1
	NIR_PBc far away (120-123)	Mobile	Embedded	1
	NIR_PBc near interface (191-194)	Mobile	Embedded	1
1KXP:D	Interface region	Rigid	-	-
	NIR_PBc far away (314-325)	Low mobility	Embedded	1
	*NIR_PBc near interface (250-258)*	*Rigid*	*-*	*-*
2BTF:A	Interface region	Rigid	-	-
	*NIR_PBc far away (156-158)*	*Rigid*	*-*	*-*
1XD3:A	Interface region	Partly mobile	Embedded	2
	*NIR_PBc near interface (89-93)*	*Rigid*	*-*	*-*
1BVK:F	Interface region	Partly mobile	Whole	1
	NIR_PBc with interface (100-105)	Mobile	Embedded	2
1B6C:B	Interface region	Rigid	-	-
	NIR_PBc near interface (15-22)	Mobile	Whole	3
	NIR_PBc near interface (152-157)	Mobile	Embedded	4
*Cases with substantial change in non-interacting residues near interacting region as well as in interacting region*				
1I2M:A	Interface region	Rigid	-	-
	NIR_PBc far away (145-149)	Mobile	Embedded	4
	NIR_PBc near interface (32-29)	Partly mobile	Embedded	2

*The abbreviations used are: *IR* - Interface regions, *NIR_PBc* – Non-interacting regions with PB change.

This table lists the results of the analysis using NMA regarding the intrinsic tendency for regions of interest to be rigid or mobile. The term ‘Embedded’ indicates that the region under consideration possesses intrinsic mobility as part of a segment, whereas the term ‘Whole’ indicates that the region is independent. NIR_PBc which are indicated to be mobile are shown in bold formatting and those indicated to be rigid are italicized. Note that the PDB codes and chains provided are for the bound form whereas the corresponding unbound form structure was used for NMA.

Apart from NMA, the extent of evolutionary conservation of these regions was also determined, using Jensen-Shannon divergence measure. For regions where normal mode analysis did not provide an indication of intrinsic motion, we analyzed whether crystal packing effects could provide an explanation for conformational changes. To determine this, symmetry-related molecules were generated for the bound and unbound molecule using PyMOL [77], and checked to find any crystal packing which could cause the change. A discussion of a few specific cases is presented below.

**Figure 6:**
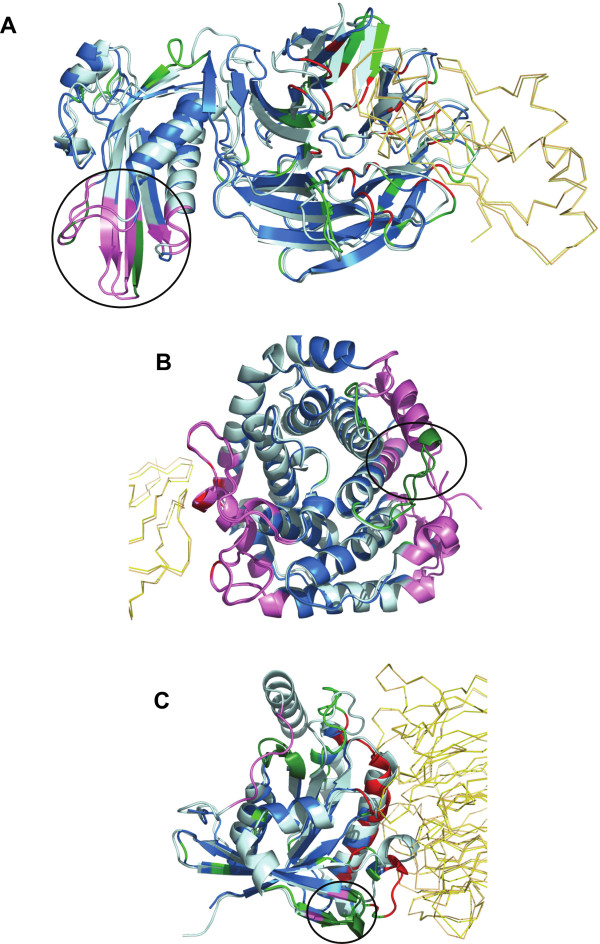
**Normal mode analysis of structural changes in regions of low B-factor away from interface.**The protein containing region of interest is depicted as cartoon and the interface of other protein in ribbon. Unbound and bound form of the protein of interest is coloured pale cyan and marine blue, respectively. The partner protein’s unbound and bound forms are coloured light orange and yellow, respectively. Interacting residues are coloured in red and non-interacting residues with PB change in green. All regions of interest are marked with a black circle, irrespective of whether they are intrinsically mobile or rigid. Regions identified to be intrinsically mobile according to NMA are coloured violet. Regions of interest occurring within the intrinsically mobile segments are coloured in dark green. The complexes shown are **A**). TolB – PAL complex (2HQS) **B**). Complement C3 and Epstein-Barr virus receptor C2 complex (1GHQ) C). Ran GTPase and Regulator of chromosome condensation (RCC1) complex (1I2M). The partner containing the region of interest is represented in italics. These figures show that noninteracting regions observed to undergo conformational changes upon complexation are usually intrinsically mobile, which is a characteristic of a functional site

a) TolB – Peptidoglycan associated lipoprotein (Pal) complex. The proteins TolB and Pal constitute the complex used by *Escherichia coli* and other ‘group A colicins’ to penetrate and kill cells [78]. TolB protein comprises of two domains – a smaller N-terminal domain and a larger C-terminal β-propeller domain which interacts with Pal protein. Large rigid-body motions and conformational changes were seen far away from the interface between the unbound and bound forms of the TolB protein (Figure 6A, encircled region). The biological relevance of these changes is supported by experiments which prove that Pal binding results in conformational changes being transmitted to N terminal α/β domain of TolB [79]. Further, a recent study shows that TolA binds to the N-terminal region exhibiting structural change in TolB [80], indicating that the structural changes occurring upon Pal binding serves as an allosteric signal. Additional support comes from GNM-based normal mode analysis of the unbound form. The region of our interest (mainly residues 86–91), was seen to have intrinsic tendency for reconfiguration in the second most significant mode pertaining to local motions (mode 3, Figure 6A, purple coloured region). However, we identify that this region is not evolutionary conserved and most of the sites occurred in the least conserved bin of residues in the protein.

b) Complement C3 and Epstein-Barr virus receptor C2 complex. Complement component C3d binds to antigenic molecules. This binding helps in further amplification of B cell responses as a consequence of the simultaneous binding of antigen-bound C3d with complement receptor type 2 and binding to B cell receptor via bound antigen [81]. Complement C3’s interaction with C2 receptor causes conformational changes, identified using PBs, at residues 264–274. No literature information specific for this region was available. However, GNM analysis of the unbound form indicated that the region near to interface and the region in the opposite side have intrinsic motion (Figure 6B, purple coloured regions). The region of our interest occurs opposite to the interface and is indicated to be partly flexible, implying that this motion could be biologically relevant (Figure 6B, green coloured segment in purple coloured region). However, this region is moderately conserved.

c) Ran GTPase and Regulator of chromosome condensation (RCC1) complex. Ran GTPase is a key component of G-protein signaling. It serves as a molecular switch which cycles between GDP- and GTP- bound states. It requires regulators for enhancing its low intrinsic hydrolysis and nucleotide dissociation rates. Guanine nucleotide exchange factors form the latter group which bind to G-proteins and induce rapid dissociation of bound GTP and hence enable fast activation to GTP bound form. The structure under consideration is a complex of Ran GTPase with the guanine nucleotide exchange factor RCC1 [82].

Ran GTPase, which adopts the P-loop containing nucleoside triphosphate hydrolases fold, contains two regions of interest, one near the interface (residues 41–44) and the other far away from the interface (residues 151–155) (Figure 6C, encircled regions). The interface also undergoes substantial changes on binding. The region of interest near the interface is seen to be intrinsically mobile in the second most important mode. The region of interest far away from the interface is seen to be a part of a mobile region in the fourth important mode. This region is very near to GDP-binding site in the unbound form. It appears that binding of Ran GTPase and RCC1 causes structural changes at distant sites (GDP-binding site) to bring about exchange of nucleotides. This case provided a clear example of signal transduction within the molecule to bring about the desired biochemical effect. Such sites can also probably be targeted by human intervention to prevent disease manifestations, such as cancer in Ran signalling pathway [83]. The sampling of homologous sequences was not diverse enough to obtain a reliable answer about its evolutionary conservation.

### Structural changes away from the interface observed largely in proteins with structurally altered interfaces

Since structural changes away from the interface appear to be common on protein binding (*n* = 34/76), we studied the structures of the proteins in the complex to understand if there are any common characteristics of the protein exhibiting these changes (target protein) versus the binding partner (effector protein).

When we analysed the type of interface (pre-made, induced-fit, other) in the target protein, we observe that pre-made interfaces constitute only 7/34 complexes undergoing structural changes away from the interface. Induced-fit and moderately induced-fit (‘other’ interfaces) constitute the bulk, accounting for 15/34 and 13/34 complexes, respectively. Allostery appears to be the most plausible explanation to connect protein binding with structural changes away from the interface, as discussed in the previous section. Therefore, most of the proteins comprising of pre-made interfaces (26/33) do not appear to be the target proteins involved in allosteric communication. On the contrary, they seem to serve as the effector molecules for transmitting the allosteric signal to the partner protein. Comparison of the global RMSDs of case 1 complexes indicates that the target proteins (Cα RMSD = 1.52 ± 0.71 Å) are significantly more flexible than their effector proteins (Cα RMSD of 0.86 ± 0.60 Å with a *p* value of 0.0018 for a Wilcoxon paired test). However, the disparity in the values could be partly caused due to the differences in lengths (effector - 183 ± 91, target - 389 ± 247, Wilcoxon paired test *p* value - 0.0006) since RMSD is dependent on the number of residues. For proteins of case 2 complexes, there is no significant variation in terms of their lengths (Effector proteins – 236 ± 105, Target proteins – 245 ± 88, Wilcoxon paired test p value – 0.73). However, the global RMSDs of the target proteins (1.65 ± 0.64) are slightly yet significantly higher than those of the effector proteins (1.04 ± 0.67) (p value – 0.0342, Wilcoxon paired test). Therefore, it appears that binding of an effector protein causes changes at the interface of the target protein that are propagated towards the distant allosteric site, providing credence to the views implicating flexibility in allosteric modulation [84].

## Discussion

Availability of bound and unbound structures of proteins provides an opportunity to address various questions regarding structural alterations occurring due to protein-protein interactions. Our study underlines that macromolecular liganded forms of proteins undergo larger structural alterations in terms of change in local conformation (captured using PBs) as well as atomic positions (captured using RMSD) compared to unliganded proteins (Figure 1). These changes are much larger than those observed due to random fluctuations characteristic of intrinsic flexibility or experimental artifacts (see Additional file 3: Figure S1).

Non-obligatory complexes occupy a niche position as key regulators of cellular homeostasis. Their specific and timely association and dissociation are crucial for bringing about required biological function. Spatial and temporal regulation of the interacting proteins is one of the ways of avoiding unsuitable complexation [85]. The other mechanism could be the use of different conformations of binding sites, which provide favourable or unfavourable binding-competence to the partner.

Transformation of binding site structure into the active form can serve as a switch to ensure correct binding at the appropriate time. Our analysis of structural alterations provides credence to this view. Surprisingly, pre-made interfaces, which are structurally invariant upon binding, shows distribution of %PB changes similar to that observed for induced-fit interfaces. This indicates that there is some extent of conformational change in all interfaces; only the nature and magnitude varies (Figure 2B). Additionally, interface of the partner of pre-made interface is usually observed to undergo significant structural changes (Figure 2A). In essence, there are no ‘completely pre-made’ interfaces in non-obligatory complexes. Crucially, significant structural changes are observed at backbone level in most of the interfaces used in this study. It is well known that side-chains undergo large structural changes upon protein-protein complexation [86]. Considered together, these results support the view that structural conformations by themselves can serve as a good mechanism to implement the required tight regulation. Lower magnitude of structural changes is generally observed to optimize complex formation, whereas larger magnitude of structural changes is observed to remove steric clashes.

We also observe a substantial proportion of instances with significant conformational changes in non-interacting regions away from the interface (Figure 4). Identification of these cases is facilitated by the ability of PBs to capture subtle structural variations. Observation of structural changes away from interface changes has been reported previously [87-89]. They could be due to various factors:

1. Flexible regions are dynamic and can take up several distinct conformations, which can have specific functional relevance [45]. Several studies have revealed that flexibility is localized to certain regions of protein structure and such dynamic sites are usually involved in both small and large molecular interaction [90,91] and enzymatic catalysis. In our study, the interface regions of TolB - Pal complex (Figure 6A) and Complement C3 - Epstein-Barr virus receptor C2 complex (Figure 6B) are shown by normal mode analysis to be intrinsically mobile.

2. Studies show that thermodynamic entropy redistribution is a common outcome of protein-protein interaction, irrespective of the net change in entropy after complexation [92]. Loss of entropy at interacting sites is many times accompanied by gain of entropy in other regions of surface. ‘Entropy-entropy compensation’ may be due to significant intermolecular motion between the interacting molecules, which recovers about half of the entropy lost due to rotational and translational components [92]. This compensatory mechanism has been postulated to be the mechanism responsible for high-specificity binding of multiple ligands at the same region of a protein.

3. The region may be functionally relevant, for e.g. a ligand/macromolecule binding site, whose conformation is regulated by an allosteric mechanism. Since binding sites are observed to be a combination of flexible and rigid sites [90], the signal based on protein-protein complexation may alter the stability and facilitate conformational change at the functionally relevant distant region. The complex of Ran GTPase with its cognate guanine nucleotide exchange factor probably utilizes this mechanism since complexation helps in altering the accessibility to the ligand on Rho protein (Figure 6C).

4. Crystallization is known to induce substantially altered conformations [44]. In our study, we ensure that this bias is accounted for (Table 3) and that the conformational changes observed are not due to such effects.

5. Trivial factors, such as missing residues near the region of interest (or) the region being near termini, could contribute to such changes [93]. Since we ruled out complexes exhibiting such changes (Table 3), the changes observed have other biological origin.

In-depth analysis of several complexes using rigorous coarse-grained NMA and literature survey indicates that a fair proportion of structural changes upon protein-protein complexation are allosteric (Figure 6, Tables 4). Such communication is largely enriched in signalling proteins, which seems plausible considering the complex regulation of signal transduction pathways achieved using the interplay of several modular elements [12]. The lesser frequency of occurrence of such changes in enzyme-inhibitor and antibody-antigen complexes is expected. In the case of the former, their interaction is usually the result of an allosteric modulation and in the latter, a very high-affinity complex is formed, which needs to be cleared.

The classical view of allostery is as a mechanism of effector binding causing functionally relevant conformational changes at a distant site [10]. The salient features of the models involves two key attributes: the presence of two conformational states of the protein, one stabilised in the unbound state and the other favoured upon binding of the allosteric effector, and induction of structural change at the target site leading to functional modulation. However, studies in the last two decades have thrown new light on this phenomenon. The observations of allosteric modulation in the absence of conformational change [94] and the introduction of allosteric perturbation in non-allosteric proteins [95] have raised the viewpoint that all dynamic proteins are possibly allosteric [96]. These studies indicate that proteins in their unbound states exist in several conformational sub-states, characterized by different population densities [97]. Allosteric perturbation results in change in the relative populations of these conformers [98]. Such studies resulted in a paradigm shift in the understanding of allostery from a structure-centric to a thermodynamics-centric phenomenon [98]. Although newer studies on allostery indicate that change in dynamics also enables allosteric communication in many cases [94-96,98,99], in this study we have confined ourselves to the study of allostery in the classical sense, as communicated by structural changes. Surprisingly, allosteric communication established only via structural changes appears to be established in almost half of the complexes upon protein binding. Consideration of dynamics along with structural changes would most probably lead to uncovering of many more protein-induced allosteric changes. Therefore, our study suggests that protein-protein binding in the case of signalling complexes, is often likely to result in downstream effects. The smaller of the two proteins in a complex, usually comprising of an unaltered interface upon protein-protein complexation, appears to be the effector molecule in most cases. The binding event generally causes changes at the interface and concomitant structural changes at the target site.

Signalling proteins are key drug targets and the usage of allosteric modulators as drugs is gaining acceptance [100,101]. In such a scenario, the understanding that most protein-protein interactions in signalling proteins are allosteric provides impetus for the design of allosteric modulators as drugs. Allosteric regulators provide certain advantages over traditional drugs, which are usually competitive inhibitors. Binding of an allosteric drug at a distant site provides reduced side-effects, saturability, modulation in the presence of true agonist etc. [84,100]. We hope that knowledge of possible allosterically modified sites identified in the signalling complexes studied in our analysis (see Additional file 10: Table S3 & Additional file 11: Table S4) serves as a starting point for combating disease manifestations.

## Conclusions

Comparison of bound and unbound structures of protein-protein complexes enables us to address various questions regarding structural alterations occurring due to interaction. Non-obligatory complexes occupy a niche position as key regulators of cellular homeostasis with appropriate and timely association and dissociation which are crucial for eliciting the necessary biological function. Structural alterations in most of the interfaces of these non-obligatory complexes support the view that conformational features by themselves can serve as a good mechanism to implement the required tight regulation.

The interface is the most altered region in the entire protein structure upon protein-protein binding, as expected. The modifications are largely conformational in nature. In the rare case of one the partners remaining unaltered, the other partner is usually observed to undergo significant structural modification, thereby supporting the ‘induced fit hypothesis’ [52] more than the ‘lock and key hypothesis’ [51].

The observation of a substantial proportion of instances with significant structural changes in non-interacting regions away from the interface implies that the binding is likely to result in downstream effects. In-depth analysis of several complexes using rigorous coarse-grained NMA and literature survey indicates that these changes have functional relevance, with most of them being allosteric. The observation of allostery-like structural changes in about half of the transient complexes suggests this phenomenon is much more prevalent in signalling complexes than appreciated before. It also appears that the reversible nature of protein-protein association and dissociation, characteristic of transient complexes, affords nature with an attractive means to bring about allostery which is generally a reversible process.

## Methods

### Datasets used

Two kinds of control datasets are used.

a) *Rigid-proteins dataset (Control dataset 1):* A dataset of 50 independently determined structures of two rigid proteins (see Additional file 1: Table S1), bovine ribonuclease (32 structures) and sperm whale myoglobin (18 structures), were taken from Rashin et.al [44]. Values calculated from this dataset for different parameters are used as thresholds to account for positional coordinate uncertainty.

b) *Monomeric-proteins dataset (Control dataset 2):* To get a general idea about the flexibility in atomic positions for a random dataset, the PDB was mined for crystal structures of proteins with the following criteria: a single chain is present in the asymmetric unit and biological unit; crystallographic resolution of the structure should be 2.5 Å or better and the structure should not contain DNA, RNA, DNA-RNA hybrid, or other ligands bound to the protein. These molecules were clustered at a sequence identity of 95% and length coverage of 100% using BLASTCLUST (http://www.csc.fi/english/research/sciences/bioscience/programs/blast/blastclust). Finally, the clusters were refined to contain only one entry for each PubmedID per cluster, which ensures that mutants are not considered, to arrive at a dataset containing 95 clusters (see Additional file 2: Table S2) of 319 independently solved protein structures.

#### Protein-protein complex (PPC) dataset

The set of curated non-obligatory protein-protein interaction complexes solved in both unbound and bound form is taken from Benchmark 3.0 dataset [34]. The set was further pruned using PISA [102] and PDB biological unit information to exclude cases containing different non-biological oligomeric forms of a protein in the unbound and bound forms (eg. X-X in unbound form and X-Y in bound form) and bound to other small ligands or peptides. All antibody-antigen complexes in the original dataset in which only the bound structure of the antibody was solved were discarded since the corresponding unbound form was not available. The final dataset consists of 76 non-obligatory complexes (see Additional file 2: Table S2). The resolution of these entries is 3.5 Å or better. Proteins in every interacting pair in the dataset is non-redundant at the level of SCOP family [47]. Although a much larger dataset can be compiled if only one of the interacting proteins is available in unbound and bound form, such a dataset was not used since our objective is to compare the changes occurring in both the proteins upon complexation.

Although our dataset is intended to contain entries of identical proteins or protein domains available in both protein-bound and free forms, practically there could be some differences in the length and region of known 3-D structures in the bound and free forms. However the overwhelming majority of the same protein available in bound and free forms have >90% sequence identity (see Additional file 2: Table S2) indicating that the bound and unbound forms are almost the same. In all the cases with % sequence identity less than 90%, it is observed that the aligned region is identical or contains very few substitutions. Further, of the 3 cases showing large length variation between the bound and unbound forms (PDB codes: 1gcq, 1qa9, 1e6j) only 1e6j features in our analysis of cases showing structural changes away from the interface. So, it appears that the analysis is robust to length variations between bound and unbound forms of a protein.

As mentioned before the dataset used in the present analysis was derived from the robust list of protein-protein complexes proposed by Weng and coworkers [34] in their protein-protein docking benchmark version 3.0. In this dataset the authors have carefully avoided the complexes with significant extent of disordered regions. Indeed in the dataset used in the current analysis none of the complex structures used has any disordered residue at the protein-protein interfaces. This could be ensured on the basis of information on missing residues given in the PDB file, by checking the distance between Cα atoms of putative adjacent residues and by checking for the presence of all the expected atoms in a residue.

### Identification of interfacial residues

If the distance between any two atoms of residues from the two proteins is less than sum of their van der Waals radii + 0.5 Å, the two residues are considered to be in the interface [53]. The van der Waals radii were taken from the literature [103].

### Classification of residues based on solvent accessibility

The residues in a structure are classified on the basis of their residue surface accessibility (RSA) which is calculated using NACCESS [104,105]. This parameter provides a normalized measure of the accessible surface area of any residue in the protein, calculated with respect to the extended form of the residue, using the NACCESS program. The cut-offs employed are: ≤5% RSA (buried residues) and ≥10% RSA (surface residues). The 5% cut-off was adopted from [106], who optimized and used it to define residues buried in monomeric proteins. Buried, surface, and interface residues constitute ~25%, 75% and 10-20% of the residues in a protein, respectively.

### Quantification of structural change

Structural change is estimated for a given residue in unbound and bound forms. A sequence alignment of the unbound and bound forms performed using CLUSTALW [107] provides the residue equivalences. Structural change is captured using two measures: RMSD and Protein Blocks. Structural change is classically captured by means of root mean square deviation (RMSD), where RMSD is calculated as follows: $RMSD=\sqrt{1/N\sum di^{2}}$ for *i* ranging from residue 1 to *n* of the dataset and *d* is the distance between N pairs of equivalent atoms. Two measures of RMSD have been employed: Cα RMSD and all-atom RMSD, based on deviation between the Cα positions of the same residue in unbound and bound forms in the former and between all-atoms of the same residue in unbound and bound forms for the latter. Deviation in side chain positions are generally expected [86] whereas large backbone changes are comparatively uncommon. Therefore, the deviation between the Cα positions of the same residue in unbound and bound form is used as an indicator of structural change mainly. The changes are captured at structural level and averaged out for the entire protein or a set of residues in a protein (for e.g. interface residues) and the averaged measures are used in the analysis. Small yet significant changes in local conformation of a protein can be captured using Protein Blocks [48]. The three dimensional structural information in the bound and unbound forms is represented in a one-dimensional form using Protein Blocks (PBs). They consist of 16 structural prototypes, each of which approximates the backbone of a five-residue peptide. Given a 3D structure, each overlapping sequence of 5-residue fragments is associated with its closest PB. The sequence of PBs is annotated in the sequence alignment obtained using CLUSTALW. Two parameters are calculated using this measure. The first parameter indicates the presence of conformational change and is calculated as % changes in PBs between unbound and bound form (PBc). The second parameter indicates the magnitude of observed change and is calculated using PB substitution score (PBSSc) for the equivalent residues.

### ‘Pre-made’ versus ‘induced-fit’ interfaces

An interface with ≥0.5 Å Cα RMSD difference between the bound and unbound forms is classified as ‘pre-made’ interface whereas an interface with ≥1.5 Å Cα RMSD difference between the bound and unbound forms is classified as an ‘induced-fit’ interface. However, there are some interfaces with lower difference in terms of magnitude but with substantial difference at the interface in comparison to the rest of the surface residues (≥10% RSA). This cut-off was chosen since 90% of the interface residues have an RSA equal to or greater than this value in the unbound form. A normalization-based metric was used to identify induced-fit interfaces exhibiting smaller structural changes as $N=\frac{C\alpha RMSD_{I}}{C\alpha RMSD_{\mathrm{ROS}}}$ where ‘CαRMSD_I_’ indicates the average Cα RMSD difference between bound and unbound form for interface, and ‘CαRMSD_ROS_’ indicates the average Cα RMSD difference between bound and unbound form for the rest of the surface.

For example, a value of two indicates a doubled change in magnitude of the interface with respect to the rest of the surface. This value is used as a cut-off to identify substantial changes localized to the interface.

### Identification of proteins with substantial structural change in non-interacting regions

To identify cases where the interface is largely invariant/moderately altered:

*Criterion 1:* Average Cα RMSD of ‘non-interacting residues with PB change’ should be ≥1 Å than the average Cα RMSD of the interface residues. For this comparison, the individual segments under consideration, were superimposed using SUPER (B.S.Neela, unpublished).

*Criterion 2:* Normalized PB substitution score of ‘non-interacting residues with PB change’ should be ≤ −2 than the normalized PB substitution score of interacting residues.

To identify cases where there are large changes at interface:

*Criterion 1:* Average Cα RMSD of both ‘non-interacting residues with PB change’ and interacting region should be ≥2 Å.

*Criterion 2:* Average Cα RMSD of both ‘non-interacting residues with PB change’ and interacting region should be > > global Cα RMSD.

### Analysis of B-factors

The B-factor (temperature factor/atomic displacement factor) of an atom reflects the degree of isotropic smearing of electron density around its center [108]. A low B-factor indicates small uncertainty in the position of an atom. A high B-factor can be caused by different factors: high thermal fluctuations, alternate conformation of an atom, and domain motion, to name a few.

To ascertain the flexibility/rigidity of a particular residue in a structure, its normalized backbone B-factor was considered [109]. Normalization with respect to all the other residues provides an idea of increase/decrease in flexibility on a standard scale. Only surface residues (≥10% RSA) were considered for the normalization since all interacting residues and non-interacting surface residues form the crux of this study. The three most N-terminal and C-terminal surface residues were excluded since their B-factors are usually high and can affect the ‘mean’ of the values. B-factors of only backbone atoms were considered as we are studying backbone changes and also since side chain are generally more flexible than backbone atoms. The normalized B-factor per residue (*B*_*i,N*_) was computed as $B_{i,N}=\frac{B_{i}-<B_{i}>}{\sigma_{\mathrm{Bi}}}$ where *B*_*i*_ is the B-factor of residue *i*, <*B*_*i*_> is the mean B-factor of the protein surface residues and σ _*Bi*_ is the s.d. for the same.

Residues with backbone B-factors ≥3, ≥2, and <1 standard deviations from the mean backbone B-factors for surface residues can be considered to have ‘very high’, ‘high’ and ‘low’ flexibility, respectively.

### Identification of regions of protein structure with intrinsic collective motions

GNM-based NMA of the unbound form of a protein was undertaken to identify intrinsic collective motions of the molecule. In this model, the biomolecule is modelled as a harmonic oscillator with every residue represented as a single site, connected by springs to nearby residues [73]. The oGNM web server [75] calculates low frequency normal modes for the unbound structure based on GNM. In GNM, the motions are isotropic by definition, thereby predicting only regions exhibiting intrinsic motion and magnitude of change. The directionality of motion cannot be predicted using GNM models. The server constructs the elastic network model of the structure by considering each of the Cα atoms as a node and identifying all interacting nodes using a distance cut-off of 10 Å. The six most low frequency modes were analyzed to check whether any of the ‘non-interacting regions with PB change’ far away from the interface show probable biologically relevant intrinsic motion.

### Determining the extent of conservation of non-interacting residues with conformational changes

xBased on the assumption that evolutionary conservation of a site in a protein family is an indicator of its functional relevance and/or structural integrity, the degree of conservation of all sites in a protein family was calculated using the Jensen-Shannon divergence measure. This metric operates on the premise that most sites in a protein family are not under any evolutionary pressure and hence have a distribution similar to background amino acid distribution. Sites under evolutionary pressure, such as functional or stabilizing sites, show amino acid distribution significantly different from the background distribution.

Homologous sequences for every protein in our PPC dataset were identified by a search employing PSI-BLAST [110] against the UNIREF90 [111] database at an e-value cutoff of 0.0001 for 3 iterations. Further, only sequences with ≥30% identity were considered. A multiple sequence alignment (MSA) of the query sequence with only the aligned regions of the homologous sequences was generated using CLUSTALW. The conservation scores for every site in the MSA was calculated using Jensen-Shannon divergence measure [112]. The sites with top 30% conservation scores are considered to be well conserved [112] and sites with bottom 30% conservation scores are considered to be poorly conserved.

### Generation of symmetry-related molecules using PyMOL

Symmetry-related molecules were generated for the bound and unbound molecule using PyMOL [77]. The crystal packing after generation of symmetry-related molecules was checked to ascertain if any crystal packing could cause the observed structural changes in a complex.

## Competing interests

The authors declare that they have no competing interests.

## Authors’ contributions

NS and AdB conceived and designed the experiments. LSS and SM performed the experiments. LSS, SM, AdB and NS analyzed the data and wrote the paper. All authors read and approved the final manuscript.

## Supplementary Material

Additional file 1 **Table S1.** List of PDB codes of structures of control datasets used in this analysis. Click here for file

Additional file 2 **Table S2.** Details of various features of PPC dataset. Click here for file

Additional file 3 **Figure S1.** Distribution of parameters capturing structural change for Control and Test datasets. Click here for file

Additional file 4 **Figure S2.** Distribution of parameters capturing structural change with respect to ‘interface area’ and ‘length of protein’. Click here for file

Additional file 5 **Figure S3.** Distribution of all-atom RMSD values for PPC dataset. Click here for file

Additional file 6 **Figure S4.** Different kinds of interfaces. Click here for file

Additional file 7 **Figure S5.** Parameters for identifying rigid-body movements. Click here for file

Additional file 8 **Figure S6.** Different types of structural changes seen at interfaces. Click here for file

Additional file 9 **Figure S7.** Distribution of parameters for interface vs. non-interacting surface regions per protein. Click here for file

Additional file 10 **Table S3.** Details of non-interacting surface residues with PB change in Case 1 complexes. Click here for file

Additional file 11 **Table S4.** Details of non-interacting surface residues with PB change in Case 2 complexes. Click here for file
