# Supplementary material for: Comparison of tertiary structures of proteins in protein-protein complexes with unbound forms suggests prevalence of allostery in signalling proteins
Source: BMC Struct Biol. 2012 May 3;12:6. doi: 10.1186/1472-6807-12-6 (PMC3427047; doi:10.1186/1472-6807-12-6)
Supplement: Additional file 1 — Table S1. List of PDB codes of structures of control datasets used in this analysis. [file 1472-6807-12-6-S1.pdf]

Table S1: List of PDB codes of structures in control datasets used in this analysis.

**Control Datasets**
Rigid-proteins dataset (Control dataset 1)
Myoglobin (18) - 1BZ6,1BZP,1BZR,1CQ2,1JP6,1L2K,1MBC,1MBD,1MBO,1SPE,1VXB,1VXD,1VXG,1YOG, 2MB5,2MYE,4MBN,5MBN
Ribonuclease A (32) - 1AFK,1AFL,1AFU,1AQP,1BEL,1EOS,1EOW,1FS3,1JVT,1JVU,1JVV,1QHC,1RBW,1RBX, 1RCA,1RNC,1RND,1RNM,1RNN, 1RNQ,1RNW,1RNX,1RNY,1RNZ,1ROB,1RUV,1XPT,3RN3,5RSA,6RSA,7RSA,9RAT
Monomeric-proteins dataset (Control dataset 2) – (95)
1BVX,1HSW,1F0W,1FLQ,1IOQ,1IR7,1JIS,8LYZ,1UIH,1LSA,1HEW,1HEL,2AUB,2C8O, 3EXD,3A3Q,1LZT,4LYM,2LYM,1LYZ,1LZA,132L,1LSM,1DKJ,1UIC,1KXW,1AT6, 1LYO,1XEI,1EY0,1IHZ,1U9R,2PW5,1STN,1KDA, 1SYC,1SNO,1P7S,1L60,2IGC,1L54,1L37,1L49,1L42,256L,1L21,1L34,3LZM,1LYD,1L02, 1L19,1L25,1L17,1L23,2LZM,1L16,1L01,171L, 189L,213L,1CKH,1JWR,1LAA,1TAY,1LZ4,1LHH,2NWD,2LHM,1LHM,1LZ1,133L,1REX, 1LOZ,2BQG,1I1Z,1EIC,1KF5,1IZP,1RTB,1RHA,1RAT,1YMN,2OP2,1RBX,3RSD,1FFA,1CUA, 1CEX,1F21,1JL1,1JXB,2RN2,2Z1G,2YV0,1RBR,1LAV,1GOA,1RDA,1KVA,1C4F,1F0B, 1JBY,1QYO,1Q4A,1RMO,1Z1P,2DUE,2Q6P,1EMA,1EMB,1BFP,1L2H,1IOB,4I1B,1S0L, 1I1B,2I1B,1HIB,9ILB,1JW4,1N3X,1OMP,1ZKB,1MPB,1ANF,2CPL,1W8V,2WLW,2DPE,2ESC, 2DSU,2DSZ,1BVV,1C5H,1HV0,1XNC,1LOH,1N7N,1FH7,2EXO,1EXP,2HIS,1QYQ,1YHH, 2HGD,2WSN,1DZO,1X6P,1H13,1XW2,2B4F,6ABP,1APB,2OL6,2OYB,1EHN,1EDQ,1NH6, 1GOK,1I1X,1SKF,1ES2,1B68,1NFN,1QK8,1O7U,1C4W,1E6M,2ID7,2CHF, 2YXF,2Z9T,3DHJ,3EKC,1YU5,2RJV,1T7A,1ZU3,2PKY,2HAD,1EDE,1BOH,2ORA,1RHS, 1BPB,1ZQW,1I5I,4GCR,1AMM,3LZ2,135L,1LMN,1LMO,1EHD,1EIS,8PTI,1BTI,1EJG, 1CRN,1F1S,1LXM,1O8I,2PEC,1JEJ,1BGT,1CEC,1CEN,4CMS,1CMS,3CMS,2FKG,2OY7, 1E0W,1V0K,1KQZ,1HVQ,2HVM,1CK3,1YT4,3DTM,1DJA,1KGE,1BLP,3BLM,1DJB, 1OXD,1QY3,1GBS,153L,1IW2,2OVA,1K5A,1UN3,1B1J,1OGW,1UBQ,1UBI, 2Q43,1XMB,1CZ1,1EQP,1KWF,1CEM,1HD8,1NJ4,1Z15,2LIV,1FLH,1PSN, 4PEP,5PEP,1PGS,1PNG,1U6D,1ZGK,2PWX,3F9E,2H2Z,2QCY,1PW2,1HCL,1MTZ, 1XQV,2Q4U,2BE4,1FQN,1HVA,1CVF,1ZSA,1JN3,1RPL,1M1H,1NPR, 1AHC,1MOM,1EUG,4EUG,1DST,1HFD,1RMM,1RRX,1YUO,1AKZ,1G6L,1LV1, 1CUS,1AGY,1PQ0,1AF3,1Q2U,1J42,1A58,1A33,1LPE,1LE4,2CY1,2CXC,1K6K,1R6C, 1PKO,3CSP,1AHQ,1CNU,2LIS,1LIS,1DC9,1IFB,1ACF,1PRQ, 1FAZ,1LWB,1LOU,2BVZ,1T2I,1BOX,1AHO,1PTX,1WY3,2F4K
