# Supplementary material for: Comparison of tertiary structures of proteins in protein-protein complexes with unbound forms suggests prevalence of allostery in signalling proteins
Source: BMC Struct Biol. 2012 May 3;12:6. doi: 10.1186/1472-6807-12-6 (PMC3427047; doi:10.1186/1472-6807-12-6)
Supplement: Additional file 3 — Figure S1. Distribution of parameters capturing structural change for Control and Test datasets. [file 1472-6807-12-6-S3.pdf]

Figure S1: Distribution of parameters capturing structural change for Control and Test datasets

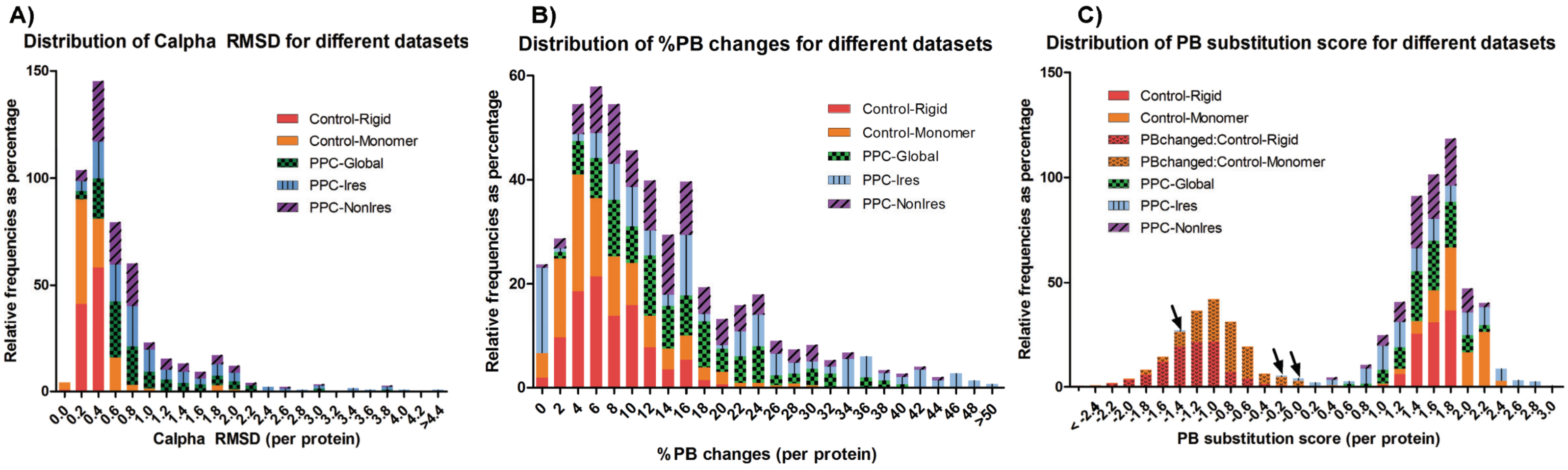

Distribution of values for the three parameters a). Cα RMSD b). %PB changes and c). PB substitution score, which quantify structural change for the following datasets: Changes in all residues (global) for Rigid, Monomeric and PPC datasets; Changes in interacting residues (Ires) for PPC dataset; Changes in Non-interacting residues (NonIres) for PPC dataset. The values plotted are calculated on a per-protein basis. In c), the arrows indicate proteins having negative PB substitutions scores for PPI-Ires. he total number of pairwise comparisons used for different datasets are: Control-Rigid (n=1328), Control-Monomer (n=2286), PPC (n=154).
