# Supplementary material for: Comparison of tertiary structures of proteins in protein-protein complexes with unbound forms suggests prevalence of allostery in signalling proteins
Source: BMC Struct Biol. 2012 May 3;12:6. doi: 10.1186/1472-6807-12-6 (PMC3427047; doi:10.1186/1472-6807-12-6)
Supplement: Additional file 4 — Figure S2. Distribution of parameters capturing structural change with respect to ‘interface area’ and ‘length of protein’. [file 1472-6807-12-6-S4.pdf]

Figure S2: Distribution of parameters capturing structural change with respect to 'interface area' and 'length of protein'

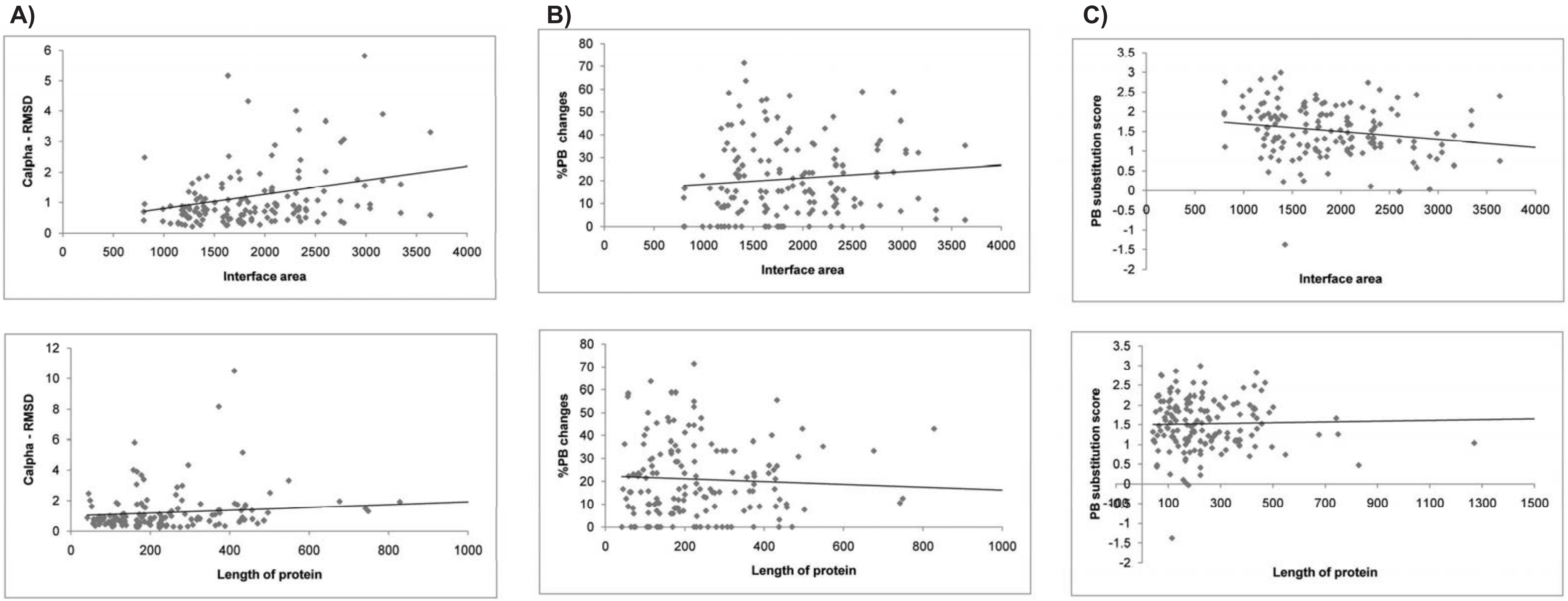

This figure shows the variation of A) Calpha-RMSD B) %PB changes and C) PB substitution score as a function of i) Interface area and ii) Length of protein. The trend-line is also indicated in the scatter plot. For the sake of clarity, a few outlier points are not shown.
