# Supplementary material for: Comparison of tertiary structures of proteins in protein-protein complexes with unbound forms suggests prevalence of allostery in signalling proteins
Source: BMC Struct Biol. 2012 May 3;12:6. doi: 10.1186/1472-6807-12-6 (PMC3427047; doi:10.1186/1472-6807-12-6)
Supplement: Additional file 5 — Figure S3. Distribution of all-atom RMSD values for PPC dataset. [file 1472-6807-12-6-S5.pdf]

Figure S3: Distribution of all-atom RMSD

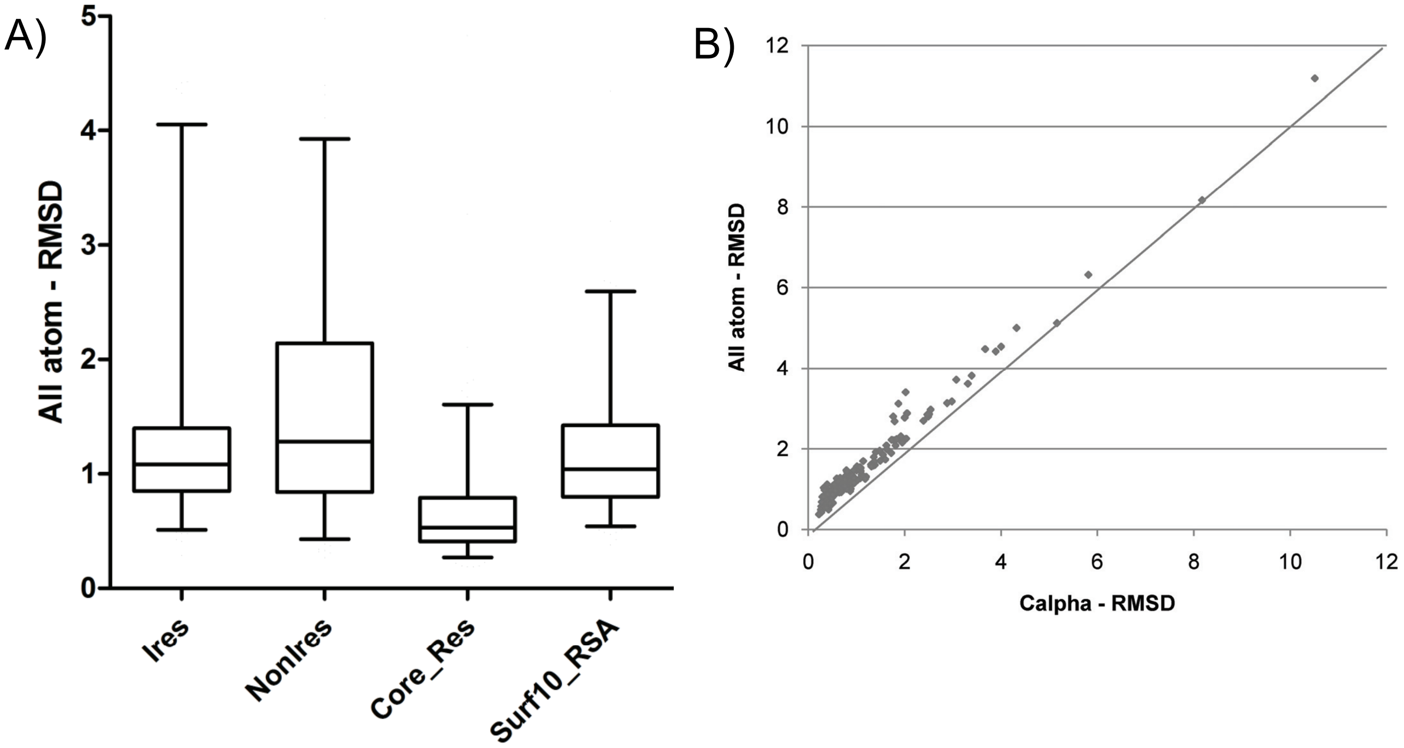

This figure shows a) The distribution of All atom – RMSD values for different residue types in the PPC dataset and b) The scatter plot depicting the correspondence between Calpha - RMSD vs. All atom – RMSD.
