# Supplementary material for: Comparison of tertiary structures of proteins in protein-protein complexes with unbound forms suggests prevalence of allostery in signalling proteins
Source: BMC Struct Biol. 2012 May 3;12:6. doi: 10.1186/1472-6807-12-6 (PMC3427047; doi:10.1186/1472-6807-12-6)
Supplement: Additional file 6 — Figure S4. Different kinds of interfaces. [file 1472-6807-12-6-S6.pdf]

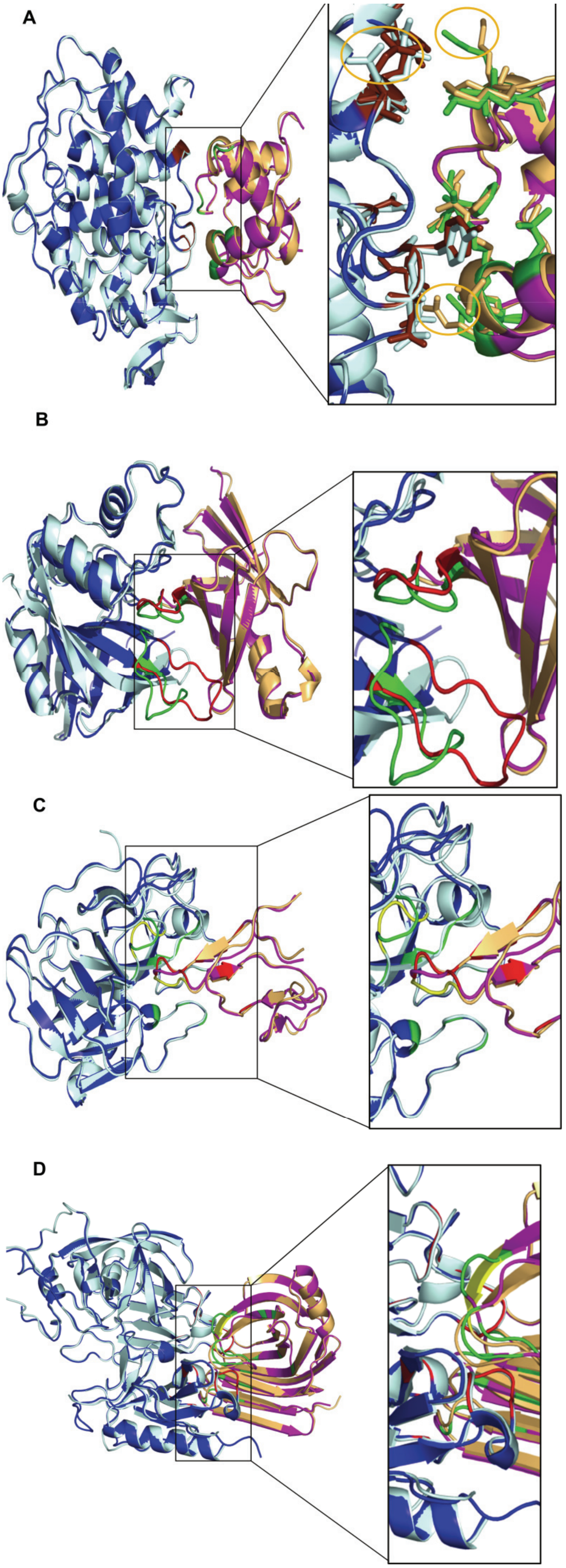

Figure S4: Different kinds of interfaces.

The unbound forms of the proteins are in light orange and light cyan and the bound form in magenta and blue, respectively. Zoomed-in pictures of the structural variations at interface are shown as inset. a). Shows a pre-made interface (cytochrome C peroxidase & iso-1-cytochrome C; 2PCC). In terms of backbone conformation, the bound and unbound forms look very similar. A zoomed-in picture of the interface depicts large changes in conformation of a few sidechains (encircled in yellow). The sidechains of the interacting residues of the two proteins are colored green and brown, respectively. b) Both proteins undergo substantial induced fit to optimise the interaction (cysteine protease & cysteine protease inhibitor; 1PXV) .The regions undergoing change are colored in green and red in the unbound and bound forms, respectively. c) Localized regions of change are seen at the interface (trypsinogen & bowman-birk proteinase inhibitor precursor; 1D6R). This case is representative of a fair population of interacting pairs. In d) structural change of smaller magnitude (~ 1Å Cα RMSD) is identified using normalization-based analysis (xylanase and xylanase inhibitor; 2B42). In both 'c' and 'd', the interacting residues are colored green and red in the two proteins. The region undergoing change is colored yellow in the unbound form.
