# Supplementary material for: Comparison of tertiary structures of proteins in protein-protein complexes with unbound forms suggests prevalence of allostery in signalling proteins
Source: BMC Struct Biol. 2012 May 3;12:6. doi: 10.1186/1472-6807-12-6 (PMC3427047; doi:10.1186/1472-6807-12-6)
Supplement: Additional file 7 — Figure S5. Parameters for identifying rigid-body movements. [file 1472-6807-12-6-S7.pdf]

Figure S5: Parameters for identifying rigid-body movements.

A)

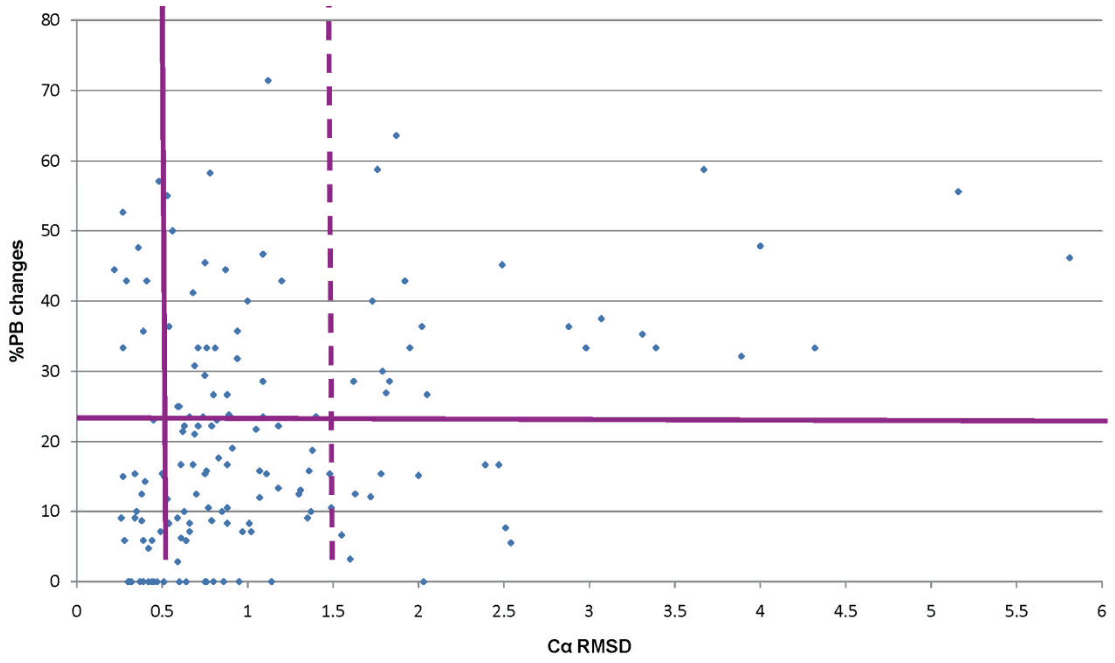

B)

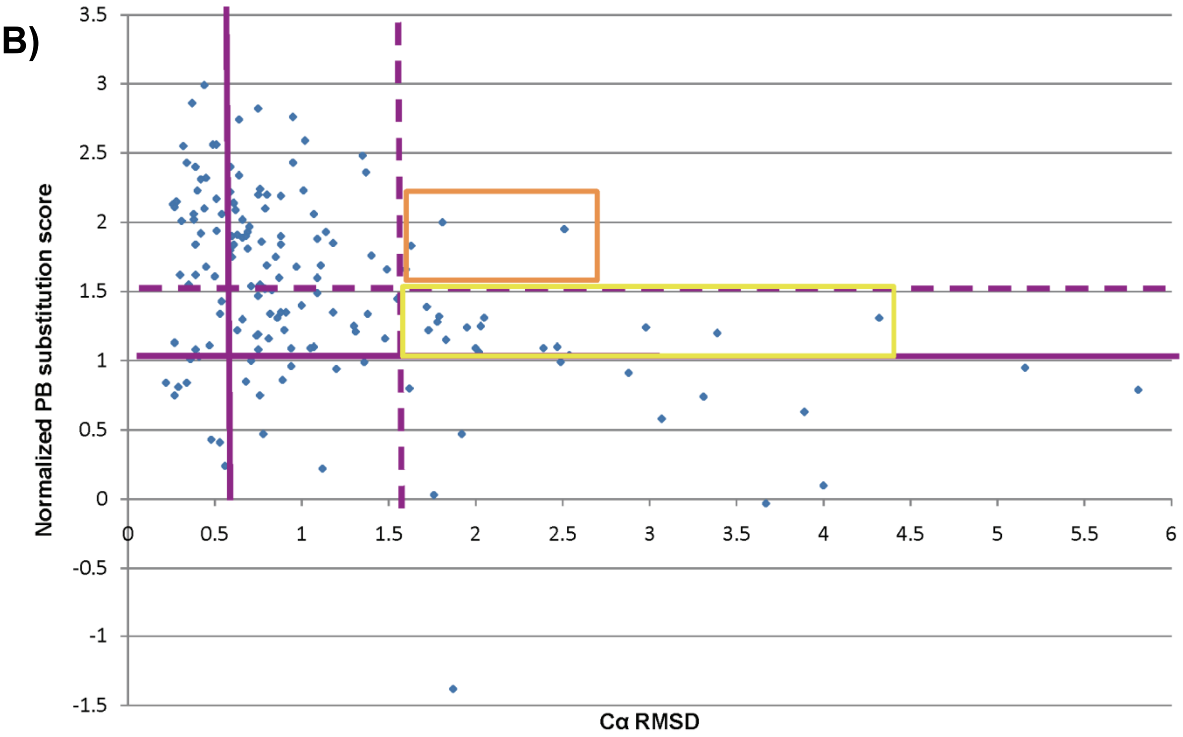

The graphs depict the information provided by considering combinations of the parameters of structural change for interfaces. Cα RMSD is plotted against a). %PB changes and b). Normalized PB substitution scores. The cutoffs derived for all the parameters from the control datasets are indicated as solid magenta lines, whereas the cutoffs indicating large changes are indicated as dashed magenta lines. Interfaces with possible large rigid body movements, characterised by Cα RMSD ≥1.5 Å and normalized PB substitution score ≥1.5 are bounded by an orange rectangle whereas probable rigid body movements, characterised by Cα RMSD ≥1.5 Å and normalized PB substitution score ≥1.0, are bounded within an yellow rectangle.
