# Supplementary material for: Comparison of tertiary structures of proteins in protein-protein complexes with unbound forms suggests prevalence of allostery in signalling proteins
Source: BMC Struct Biol. 2012 May 3;12:6. doi: 10.1186/1472-6807-12-6 (PMC3427047; doi:10.1186/1472-6807-12-6)
Supplement: Additional file 8 — Figure S6. Different types of structural changes seen at interfaces. [file 1472-6807-12-6-S8.pdf]

Figure S6: Different types of structural changes seen at interfaces.

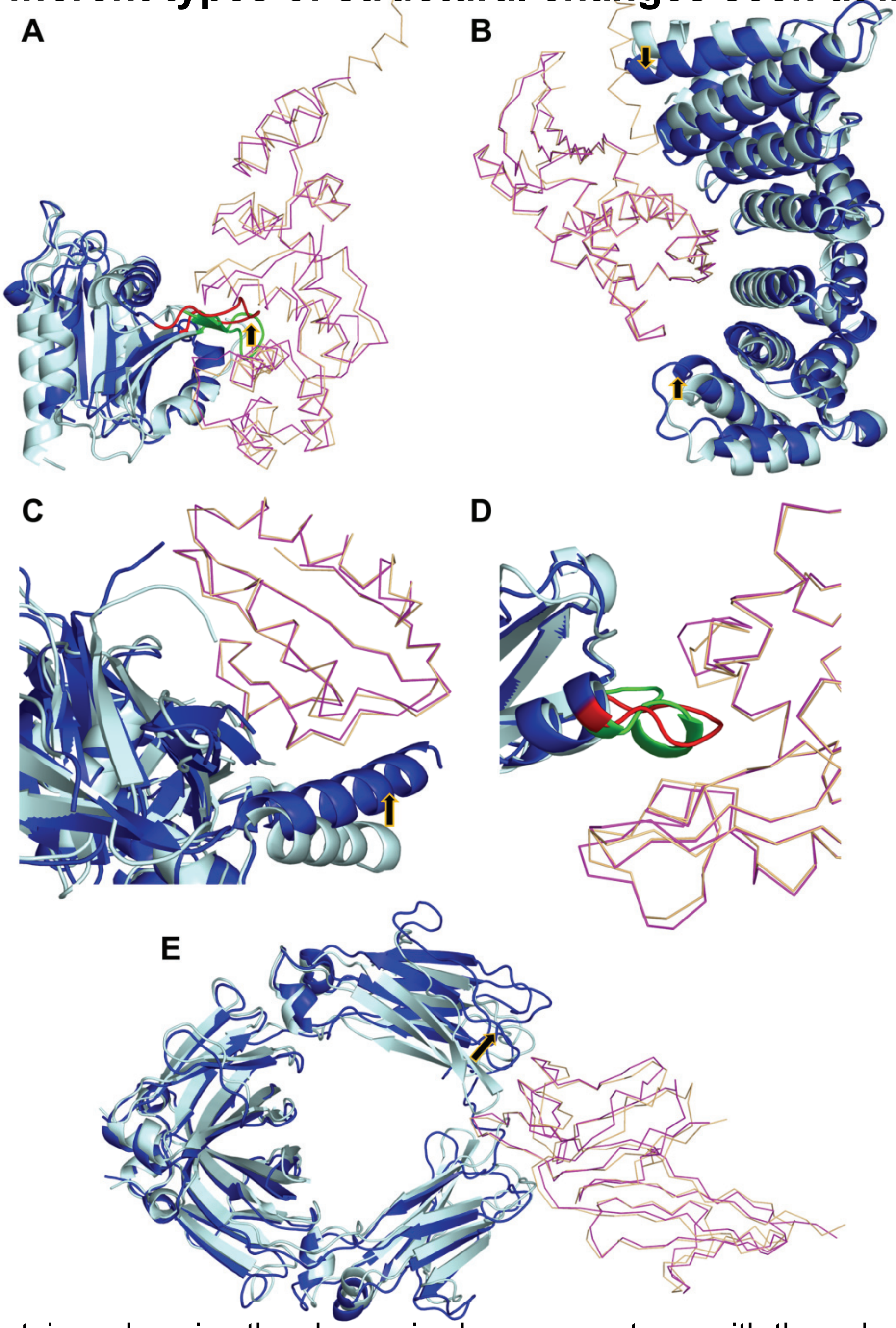

In all cases, the protein undergoing the change is shown as cartoon, with the unbound form in light cyan and the bound form in blue, and its partner as a ribbon, with the unbound form in light orange and the bound form in magenta. Figures b,c,e depict different kinds of rigid body movements with differing functionalities. Figures a,d show the two types of conformational changes usually observed at interfaces. a) Conformational change is accompanied with movement to avoid steric clash (ADP ribosylation factor & Arno; 1R8S). b). Entire protein *moves in* from all sides to optimise the interaction (Heat shock protein 1 & HSPBP1 protein; 1XQS). c) Case of local rigid body movement (encircled in yellow) of a helix towards the interacting partner to optimise the interaction (phosphocarrier protein HPR & HPR kinase/phosphorylase; 1KKL). d) Conformational change to optimise the interaction (Lysozyme C & New antigen receptor PBLA8; 2I25). e) Domain containing one of the interacting regions *moves out* to avoid a steric clash (Fc fragment of human IgG1 & Immunoglobulin gamma Fc receptor III; 1E4K). In figures a,d the region of interest is colored green and red in the unbound and bound forms, respectively. In all figures, the direction of movement is indicated by a black arrow.
