# Supplementary material for: Comparison of tertiary structures of proteins in protein-protein complexes with unbound forms suggests prevalence of allostery in signalling proteins
Source: BMC Struct Biol. 2012 May 3;12:6. doi: 10.1186/1472-6807-12-6 (PMC3427047; doi:10.1186/1472-6807-12-6)
Supplement: Additional file 9 — Figure S7. Distribution of parameters for interface vs. non-interacting surface regions per protein. [file 1472-6807-12-6-S9.pdf]

Figure S7: Distribution of parameters for interface vs. non-interacting surface regions per protein.

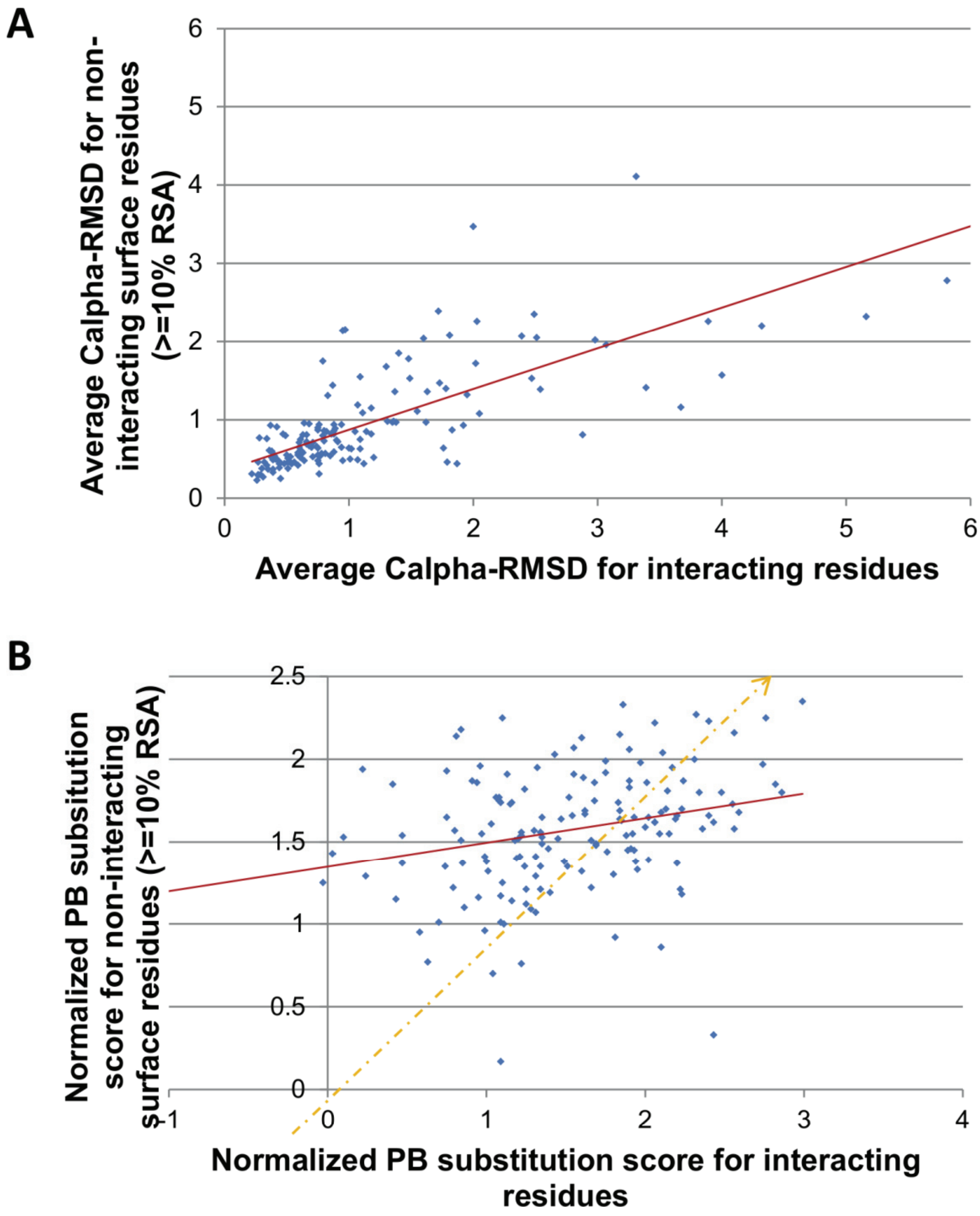

Scatter plot of a). Cα RMSD b). PB substituion score for interacting residues vs. rest of surface residues for each of the 77 protein-protein complex pairs. The line indicating the trend of the scores is shown in brown. The line depicting an exact 1:1 correspondence in scores is shown as an yellow dotted line.
